# Identifying older adults’ communication support needs and preferences: a scoping review of measurement instruments

**DOI:** 10.1093/geront/gnag033

**Published:** 2026-04-07

**Authors:** Asmita V Manchha, Bridget Burton, Michelle King, Chloe Tanswell, Samantha Siyambalapitiya, Joanne M Wood, Louise Hickson, Deirdre Fetherstonhaugh, Kirstine Shrubsole, Geoff Argus, Nerina Scarinci, Sarah J Wallace

**Affiliations:** Queensland Aphasia Research Centre, School of Health and Rehabilitation Sciences, The University of Queensland, Brisbane, Queensland, Australia; STARS Education and Research Alliance, Surgical Treatment and Rehabilitation Service (STARS), The University of Queensland and Metro North Health, Brisbane, Queensland, Australia; Queensland Aphasia Research Centre, School of Health and Rehabilitation Sciences, The University of Queensland, Brisbane, Queensland, Australia; STARS Education and Research Alliance, Surgical Treatment and Rehabilitation Service (STARS), The University of Queensland and Metro North Health, Brisbane, Queensland, Australia; Queensland Aphasia Research Centre, School of Health and Rehabilitation Sciences, The University of Queensland, Brisbane, Queensland, Australia; STARS Education and Research Alliance, Surgical Treatment and Rehabilitation Service (STARS), The University of Queensland and Metro North Health, Brisbane, Queensland, Australia; Queensland Aphasia Research Centre, School of Health and Rehabilitation Sciences, The University of Queensland, Brisbane, Queensland, Australia; STARS Education and Research Alliance, Surgical Treatment and Rehabilitation Service (STARS), The University of Queensland and Metro North Health, Brisbane, Queensland, Australia; School of Health Sciences and Social Work, Griffith University, Gold Coast, Queensland, Australia; Optometry and Vision Science, School of Clinical Sciences, Queensland University of Technology, Brisbane, Queensland, Australia; School of Health and Rehabilitation Sciences, The University of Queensland, Brisbane, Queensland, Australia; Centre for Hearing Research (CHEAR), School of Health and Rehabilitation Sciences, The University of Queensland, Brisbane, Queensland, Australia; Australian Centre for Evidence Based Aged Care (ACEBAC), La Trobe University, Melbourne, Victoria, Australia; Queensland Aphasia Research Centre, School of Health and Rehabilitation Sciences, The University of Queensland, Brisbane, Queensland, Australia; STARS Education and Research Alliance, Surgical Treatment and Rehabilitation Service (STARS), The University of Queensland and Metro North Health, Brisbane, Queensland, Australia; Southern Queensland Rural Health, The University of Queensland, Toowoomba, Queensland, Australia; School of Health and Rehabilitation Sciences, The University of Queensland, Brisbane, Queensland, Australia; Centre for Hearing Research (CHEAR), School of Health and Rehabilitation Sciences, The University of Queensland, Brisbane, Queensland, Australia; Queensland Aphasia Research Centre, School of Health and Rehabilitation Sciences, The University of Queensland, Brisbane, Queensland, Australia; STARS Education and Research Alliance, Surgical Treatment and Rehabilitation Service (STARS), The University of Queensland and Metro North Health, Brisbane, Queensland, Australia

**Keywords:** Communication, Measurement instrument, Aging, Communication support needs, Aged care

## Abstract

**Background and Objectives:**

Communication changes in older adults affect independence, social participation, and quality of life. In aged care settings, timely identification of communication support needs and preferences is essential to upholding older people’s rights and ensuring person-centered care, yet communication is not routinely assessed. This scoping review systematically identifies and describes instruments that measure communication support needs and preferences in older adults.

**Research Design and Methods:**

Following Preferred Reporting Items for Systematic Reviews and Meta-Analyses extension for Scoping Reviews guidelines, we searched PubMed, PsycINFO, CINAHL, and Embase alongside gray literature. Titles, abstracts, and full texts were screened against eligibility criteria using Covidence. Data on instrument context, content, and administration were extracted and synthesized using content analysis and the World Health Organization International Classification of Functioning, Disability and Health framework.

**Results:**

26 publications describing 29 instruments were included. Instruments were typically condition-specific (e.g., dementia) or impairment-specific (e.g., hearing loss). Communication preferences and personal factors, such as cultural and linguistic background, were rarely considered, and only one instrument assessed both needs and preferences. While self-report instruments were often intended for completion by the older adult, only one offered a communication-accessible version (e.g., easy read text, larger font, visual aids).

**Discussion and Implications:**

No existing instrument adequately measures communication support needs and preferences for use in aged care settings. A condition-agnostic instrument is needed that adopts a holistic, biopsychosocial perspective; accounts for the aged care environment; incorporates multiple perspectives; and is communication-accessible. Developing such a measure is a foundational step toward accurate, consistent, and timely identification of the communication needs of older adults in aged care.

## Background

Many older adults experience changes to their communication that impact participation in everyday social and care-related activities (Cummings, 2023). The changes to communication experienced by older adults are diverse and can result from typical aging processes (e.g., age-related hearing loss), as well as a higher prevalence of diseases that impact communication (e.g., dementia) (Yorkston et al., 2010). Communication is often further impacted by personal (e.g., language spoken and culture) and contextual (e.g., communication partner skills and the physical environment) factors (Manchha et al., 2025). Recent initiatives, such as the *Gerontological Society of America’s* Communication Strategies for Older Adults guide (2025), have highlighted evidence-based approaches and best practices for fostering clearer, more respectful communication between older adults and their care providers, emphasizing the importance of adapting communication environments and practices to individual needs and preferences. The identification of communication needs and preferences is important for supporting quality of life in older adults (Krein et al., 2019). “Communication support needs” recognizes the additional support and/or interventions that individuals require due to motor, sensory, cognitive, emotional, or behavioral difficulties, or because of physical and/or social barriers to communication in the person’s environment (Speech Pathology Australia, 2024). “Communication preferences” are defined as favorable, frequently used, and familiar methods of communicating (Yuan et al., 2016) such as using interpreter services, using video rather than voice calls, or preferring to talk in small, rather than large groups. Improved communication is an identified priority for older individuals (Australian Older Persons Advocacy Network [OPAN], 2024), yet there is limited understanding of how support needs and preferences are identified and documented in aged care settings.

Communication is a foundational human right, and an enabler of other rights. Rights-based understandings and approaches have been slower to develop for older adults than for other groups, such as children or people with disabilities. The expansion of rights-based approaches for older people also underlies the provision and delivery of state-funded aged care services, seen in Australia’s new *Aged Care Act 2024* (Cth) which explicitly includes communication in several of its “Statement of Rights” (Pt. 3, Div. 1). Within these recent human rights-based approaches to aged care delivery and the treatment of older persons, there is considerable uncertainty in how a “right” to communication, or a “right” to support for communication may be enlivened meaningfully. Without clarity of what a “right” to communication may entail in itself, it is difficult to determine how communication may enable other rights, such as the right to support for decision-making (Aged Care Act 2024, Pt. 3, Div. 1, s. 23 (7)–(9)) or the right to make complaints which are resolved fairly (Pt. 3, Div. 1, s. 23). How governments, funding bodies, regulators, and service providers can uphold a legislated “right” to communication for older adults (or other rights which communication enables) is a matter of considerable uncertainty (Mackay et al., 2023). It must, however, begin with the identification of those people who have a need for communication support. The current review was conducted in order to identify and describe measurement instruments that could be used to identify older adults’ communication support needs and preferences.

We employed the World Health Organization International Classification of Functioning, Disability and Health (ICF, WHO, 2001) as a framework for this review. The ICF was chosen as an interdisciplinary, standardized framework, which can capture the full spectrum of human functioning and disability. This framework recognizes that functioning and disability occur in context and is the outcome of interactions between (a) *body functions*- physiological functions of body systems (e.g., sensory impairments), (b) *activities and participation*—an individual’s ability to perform tasks (e.g., reading), with a focus on the restrictions or barriers they may face in doing so, (c) *environmental factors—*facilitators/barriers to functioning from the physical, social, and attitudinal environment (e.g., support/relationships) and (d) *personal factors*—facilitators/barriers to functioning from characteristics and background (e.g., language spoken). In its biopsychosocial assessment of functioning and disability, the ICF aligns with a person-centered approach to aging, recognizing that communication is the result of dynamic interactions between a person, their health condition, and the environment in which they live (WHO, 2002). It was therefore considered a suitable framework for understanding how the communication needs and preferences of older people are currently measured. The specific aims of this review were to identify and describe instruments that measure the communication support needs and preferences of older adults who receive aged care services, including (a) the context in which they are designed to be used, (b) constructs measured, and (c) modes of administration.

## Method

### Design

A scoping review methodology (Munn et al., 2022) was selected for systematic identification and synthesis of available literature, across a wide range of evidence sources. This review was designed and conducted in alignment with the Preferred Reporting Items for Systematic Reviews and Meta-Analyses extension for Scoping Reviews (PRISMA-ScR; Tricco et al., 2018) guidelines and was registered on Open Science Framework (https://doi.org/10.17605/osf.io/ubk4x).

### Research question

Our research question was, “what observational and self-report measurement instruments exist to profile the communication support needs and preferences of older adults who receive aged care services?” Subquestions were (a) in what contexts are they used? (b) what constructs do they measure? and (c) how are they administered?

### Consumer and community involvement

This review is part of a larger program of research that aims to improve communication in aged care settings. The project is overseen by a Lived/Living Experience Advisory Group (LEAG) consisting of six stakeholders with personal experience of aged care services, including people receiving aged care services (*n* = 3), family members/significant others of people receiving aged care services (*n* = 2), and aged care staff (*n* = 1). The LEAG supported the development of the search strategy by reviewing and adding to the search terms (e.g., types of communication support needs and/or preferences). Members also identified peak body/industry groups (e.g., Older Person’s Advocacy Network) that could support identification of relevant measurement instruments.

### Information sources and search strategy

A search strategy was designed based on Manchha et al. (2025) and Krein et al. (2019) and in consultation with the research team, a health science librarian, members of the LEAG, and aged care providers/industry organizations. It considered (a) the population—older adults (i.e., people aged 65 and over, 55 and over for First Nations people) and/or aged care recipients, (b) the phenomenon of interest—self-report and/or observational measurement instruments, and (c) the context—physical and/or social facilitators/barriers to communication. An example of the search strategy is presented in Table 1, with complete versions available in Supplementary Materials (S1–S5).

**Table 1:** Example search strands.

Search	Construct/s	Search strands
Set 1	Older adults and/or aged care recipient	“older adult*”[Title/Abstract] OR “older person*”[Title/Abstract] OR “older people”[Title/Abstract] OR “elder*”[Title/Abstract] OR “aged care resident*”[Title/Abstract] OR “aged care recipient*”[Title/Abstract] OR “aged care client*”[Title/Abstract] AND “aged”[Title] OR “elder*”[Title] OR “aged care”[Title/Abstract] OR “aged-care”[Title/Abstract] OR “rest home”[Title/Abstract] OR “care home”[Title/Abstract] OR “aged care context”[Title/Abstract] OR “aged care setting”[Title/Abstract] OR “older adult care”[Title/Abstract] OR “older adult nursing”[Title/Abstract] OR “gerontolog*”[Title/Abstract] OR “geriatric*”[Title/Abstract] OR “elder care”[Title/Abstract] OR “aged care nursing”[Title/Abstract] OR “skilled nursing facilit*”[Title/Abstract] OR “care facilit*”[Title/Abstract] OR “institutionalized elder*”[Title/Abstract] OR “institutionalised elder*”[Title/Abstract] OR “gerontologic nursing”[Title/Abstract] OR “residential aged care”[Title/Abstract] OR “residential care”[Title/Abstract] OR “long term care”[Title/Abstract] OR “long-term care”[Title/Abstract] OR “nursing care facilit*”[Title/Abstract] OR “old age home”[Title/Abstract] OR “nursing home*”[Title/Abstract] OR “assisted living facilit*”[Title/Abstract] OR “home for the aged”[Title/Abstract] OR “housing for the elderly” “community aged care”[Title/Abstract] OR “retirement village”[Title/Abstract] OR “at-home care”[Title/Abstract] OR “health services for the aged”[Title/Abstract] OR “geriatric health services”[Title/Abstract] OR “health services for the elderly”[Title/Abstract] OR “geriatric health service”[Title/Abstract]
Set 2	Measurement instrument	“screening instrument”[Title/Abstract] OR “checklist”[Title/Abstract] OR “assessment”[Title/Abstract] OR “tool*”[Title/Abstract] OR “needs assessment”[Title/Abstract] OR “instrument”[Title/Abstract] OR “screening”[Title/Abstract] OR “evaluation”[Title/Abstract] OR “scale”[Title]
Set 3	Communication support needs and/or preferences	“communication support needs”[Title/Abstract] OR “communication need*”[Title/Abstract] OR “communicat*”[Title/Abstract] OR “conversation”[Title/Abstract] OR “talk*”[Title/Abstract] OR “speech”[Title/Abstract] OR “speak*”[Title/Abstract] OR “communication barrier*”[Title/Abstract] OR “miscommunication”[Title/Abstract] OR “communication breakdown”[Title/Abstract] OR “misunderstanding”[Title/Abstract] OR “language impairment*”[Title/Abstract] OR “language disorder”[Title/Abstract] OR “Communication Aids for Disabled”[Title/Abstract] OR “communication support*”[Title/Abstract] OR “communication aid”[Title/Abstract] OR “communication management”[Title/Abstract] OR “communication strategy”[Title/Abstract] OR “communication strategies”[Title/Abstract] OR “communication disability*”[Title/Abstract] OR “communication difference*”[Title/Abstract] OR “communication impairment*”[Title/Abstract] OR “communication difficult*”[Title/Abstract] OR “communication issue*”[Title/Abstract] OR “communication disorder*”[Title/Abstract] OR “dementia” OR “cognitive impairment” OR “vision impairment” OR “hearing impairment” OR “voice impairment” OR “speech impairment” OR “language” OR “neuropsychiatric” OR “hearing loss” OR “visually impaired persons” OR “mental health” OR “blind people*” OR “deaf people” OR “persons with hearing impairments” OR “intergenerational”[Title/Abstract] OR “Vulnerable population*”[Title/Abstract] OR “minority group*”[Title/Abstract] OR “culturally responsive”[Title/Abstract] OR “cultural*”[Title/Abstract] OR “ethnic*”[Title/Abstract] OR “cross cultural”[Title/Abstract] OR “minority”[Title/Abstract] OR “racial”[Title/Abstract] OR “linguistically diverse”[Title/Abstract] OR “culturally diverse”[Title/Abstract] OR “CALD”[Title/Abstract] OR “linguistic diversity”[Title/Abstract] OR “bilingual”[Title/Abstract] OR “bi lingual”[Title/Abstract] OR “bi cultural”[Title/Abstract] OR “bicultural”[Title/Abstract] OR “multilingual”[Title/Abstract] OR “multi lingual”[Title/Abstract] OR “cross cultural care”[Title/Abstract] OR “ethno specific”[Title/Abstract] OR “cultural diversity”[Title/Abstract] OR “multicultural*”[Title/Abstract] OR “multi cultural*”[Title/Abstract] OR “immigrant”[Title/Abstract] OR “migrant”[Title/Abstract] OR “gender differences”[Title/Abstract] OR “digital illiteracy”[Title/Abstract] OR “limited english”[Title/Abstract] OR “english as second language”[Title/Abstract] OR “Refugee*”[Title/Abstract] OR “lgbt*”[Title/Abstract] OR “LGBT*”[Title/Abstract] OR “gay”[Title/Abstract] OR “lesbian*”[Title/Abstract] OR “bisexual*”[Title/Abstract] OR “queer”[Title/Abstract] OR “transgender”[Title/Abstract] OR “non-binary”[Title/Abstract] OR “non binary”[Title/Abstract] OR “indigenous people*”[Title/Abstract] OR “aborigin*”[Title/Abstract] OR “indigenous”[Title/Abstract] OR “torres strait*”[Title/Abstract] OR “first nation*”[Title/Abstract] OR “first people*”[Title/Abstract] OR “financially disadvantaged”[Title/Abstract] OR “remote population*”[Title/Abstract] OR “isolated communit*”[Title/Abstract] OR “veteran*”[Title/Abstract] OR “physical environment”[Title/Abstract] OR “support person*”[Title/Abstract] OR “communication style”[Title/Abstract] OR “relationships”[Title/Abstract] OR “communication device*”[Title/Abstract] OR “communication aid*”[Title/Abstract] OR “technology”[Title/Abstract] OR “health service*”[Title/Abstract] OR “work design”[Title/Abstract] OR “staff*”[Title/Abstract] OR “built environment”[Title/Abstract] OR “social environment”[Title/Abstract] OR “environment design”[Title/Abstract]) AND (“aged care”[Title/Abstract] OR “aged-care”[Title/Abstract] OR “rest home”[Title/Abstract] OR “care home”[Title/Abstract] OR “aged care context”[Title/Abstract] OR “aged care setting”[Title/Abstract] OR “older adult care”[Title/Abstract] OR “older adult nursing”[Title/Abstract] OR “gerontolog*”[Title/Abstract] OR “geriatric*”[Title/Abstract] OR “elder care”[Title/Abstract] OR “aged care nursing”[Title/Abstract] OR “skilled nursing facilit*”[Title/Abstract] OR “care facilit*”[Title/Abstract] OR “institutionalized elder*”[Title/Abstract] OR “institutionalised elder*”[Title/Abstract] OR “gerontologic nursing”[Title/Abstract] OR “residential aged care”[Title/Abstract] OR “residential care”[Title/Abstract] OR “long term care”[Title/Abstract] OR “long-term care”[Title/Abstract] OR “nursing care facilit*”[Title/Abstract] OR “old age home”[Title/Abstract] OR “nursing home*”[Title/Abstract] OR “assisted living facilit*”[Title/Abstract] OR “home for the aged”[Title/Abstract] OR “housing for the elderly” “community aged care”[Title/Abstract] OR “retirement village”[Title/Abstract] OR “at-home care”[Title/Abstract] OR “health services for the aged”[Title/Abstract] OR “geriatric health services”[Title/Abstract] OR “health services for the elderly”[Title/Abstract] OR “geriatric health service”[Title/Abstract]

Four electronic databases (PubMed, PsycINFO CINAHL, Embase) and gray literature (using Google Advanced search and webpages from professional organization websites, S1), were searched. Searches were restricted to items published in English up until June 2024. We further identified relevant publications from the reference lists of papers included in the review and in consultation with the research team who have expertise in aged care, nursing, speech pathology, audiology, optometry, and culturally responsive health care.

### Inclusion and exclusion criteria

We used the following inclusion criteria (Table 2) to select evidence sources (a) published in English; (b) published as a peer-reviewed journal article, book, or webpage (identified via Advanced Google search); (c) reports the use, development, or validation of a measurement instrument for profiling communication support needs or preferences in older adults; and (d) reports a measurement instrument which is self- or observer-reported. Self-report measurement instruments (also known as patient-reported outcome measures) use a report that comes directly from the patient or participant without interpretation from others, for example, a questionnaire or interview completed by an older person (NCATS, 2026a). Observer-reported measurement instruments use reporting based on observable signs, events, or behaviors and are completed by someone other than the patient/participant (NCATS, 2026b). For the purposes of this review, we further divided this category to describe those instruments intended to be completed by (a) family/close caregivers and (b) professionals. Professionals were further described in terms of being specialists (i.e., where specialist clinical interpretation is needed to complete a measurement instrument) or non-specialist workers.

**Table 2:** Eligibility criteria.

Source	Included	Excluded
Publications	Written in English empirical studies including quantitative, qualitative, mixed methods Peer reviewed, published journal articles, books and book chapters, webpages	Articles in a language other than English Theoretical papers, commentary, opinion/editorials, protocol papers, reviews Unpublished articles Book reviews Journal articles not peer reviewed Gray literature: magazines, letters, interviews, gov reports, theses, conference papers
Set 1: Older adults and/or aged care recipient	People aged 65 or older (55+ for First Nations people) Aged care recipient	Younger people receiving care (<65/55 years)
Set 2: Measurement instrument	Observational and/or self-report measurement instrument (e.g., screening assessment, tool, assessment, measure, outcome measure, profile, evaluation) that aims to identify communication support needs and/or preferences.	Performance-based or a laboratory-based measures. Version in language other than English with no translation available Measurement instrument with items not relevant to communication Measurement instrument not specifically intended for older adult population
Set 3: Communication support needs and/or preferences.	*Needs of older adults who require additional support and/or intervention with their communication skills* Communication disabilities, impairment*, communication difficulties, communication issues, communication disorder Communication (including Speech*, Language, Talk*, Speak*, Express*, Interact*, Conversation, “verbal,” “nonverbal”) **+** *Health* (including hearing impairment, vision impairment) *Personal* (including communication differences/culturally responsive communication practices, language barriers, culturally diverse backgrounds, immigrants/migrant, refugees, gender, sexuality, first persons, intergenerational backgrounds) *Environmental factors* (including psychosocial conditions and aged care)	Studies that did not include communication practices & descriptions of communication Studies that did not discuss how communication impacted older adults’ ability to participate fully in everyday activities/conversations Studies describing content/frequency of communication, development of a communication device, social/therapy/educational intervention without describing a measurement instrument

### Screening and data extraction

Searches were conducted in July 2024. Covidence online software (https://www.covidence.org/) was used to manage search results, remove duplicates, and screen titles, abstracts, and full texts. Two reviewers independently screened titles, abstracts, and full texts. Reviewer 1 (A.V.M.), an aged care researcher, drew on her lived experience of research in aged care systems to identify how contextual factors may impact older adults’ communication, whereas Reviewer 2 (C.T.), a certified practicing speech pathologist, provided insight from a clinical perspective regarding communication disability. Both reviewers independently screened all titles and abstracts against pre-determined eligibility criteria. Reviewer 1 screened all full text sources. Reviewer 2 screened a sub-sample of approximately 30% of full text sources, with an inter-rater reliability Kappa indicating 0.80 (strong agreement). Publications that appeared as “conflicts” were cross-examined and discussed with the research team until a decision about inclusion was reached. The first level of data extraction involved the identification of relevant measurement instruments. Publications reporting the same measurement instrument were grouped together. Secondary searches were conducted to find full versions of identified measurement instruments. Data were then extracted to describe each instrument (name, original/secondary source, area of communication measured, number of items/questions, response type/rating scale, administration, target audience, administrator, duration, context, study design). A qualitative content analysis approach was used to identify the main concepts measured by each instrument at an item level. These concepts were subsequently classified according to ICF domains. Data were extracted by the first author (who was also the first reviewer) and checked by the second reviewer (C.T.), and reviewed by the project team. In alignment with the review aims, extracted data were tabulated to demonstrate: target population and setting; ICF domains and primary constructs measured, administrator requirements (specialist or non-specialist), administration time and the method of report (self- or observer-reported); and availability of language translations or communication-accessible versions (i.e., versions using simplified language, visual aids, and specific formatting intended to support comprehension). Each instrument was then considered in terms of its potential for use within aged care environments, with consideration of: *relevance–*the extent to which instruments measure factors known to influence communication in older adults; *usability—*suitability for routine use in aged care settings; and *equity—*accessibility for diverse populations. A quality assessment of included sources was not conducted, in line with scoping review practice (Xie et al., 2024).

## Results

### Search results

**Figure 1:**
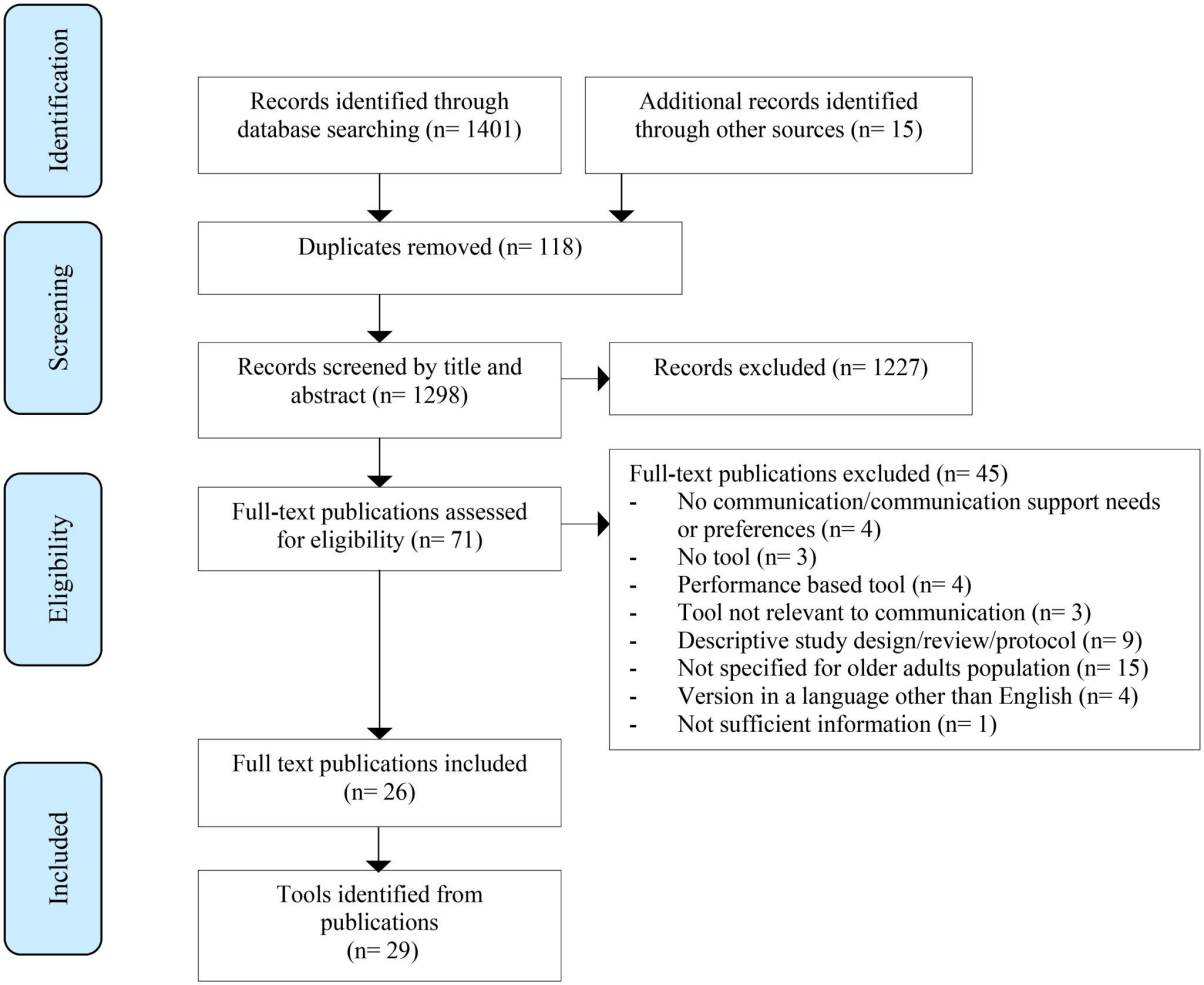
PRISMA-ScR chart.

A total of 1,415 publications were identified, 1,401 from database searching and 15 from manual searches. From these, 118 duplicates were removed, and the remaining 1,298 publications were screened for eligibility based on title and abstract. Following full-text review screening, 1,272 publications were excluded, and 26 publications were included in line with our eligibility criteria (see Figure 1; PRISMA-ScR Chart).

From these 26 publications, 29 measurement instruments were identified and included in the data extraction phase (see Table 3). A comprehensive description of each measurement instrument is provided in the Supplementary Materials (S2–S4).

**Table 3:** Summary of identified measurement instruments (*n = *29).

Measurement instrument	Citation of measurement instrument	Context		Content		Versions	Administration	
		Intended population/condition	Developed specifically for older adults or aged care settings?	ICF domains measured	Main communication-related constructs measured		Administration time	Method of report
1. ASHA Functional Assessment of Communication Skills for Adults	Frattali et al. (2017)	Adults with neurologically-based communication disorders	N	A&P	Functional communication; social communication; communication of basic needs; reading, writing, and number concepts, and daily planning.	Available in English, Chinese, Italian, Portuguese	20 minutes	Clinician-rated (specialist). Likert-scale ratings based on speech pathologists’ observations.
2. ASHA Quality of Communication Life Scale	Paul et al. (2004)	Adults with neurologically-based communication disorders	N	QOL^a^	Communication-related quality of life: confidence and autonomy in communication, roles and self, participation in daily activities, interaction with others.	–	15 minutes	Self-report. Rating scales completed by person with communication disorder.
3. Behavioral Assessment Scale (BAS)—Communication subscale	Ritchie and Ledesert (1991)	Older adults with dementia	Y	BF, A&P	Verbal comprehension and expression, nonverbal expression, written comprehension and expression, social interaction.	Available in French		Clinician-rated (non-specialist) scale.
4. Camberwell Assessment of Need for the Elderly (CANE)	Reynolds et al. (2000)	Older adults with mental illness	Y	BF	Vision, hearing.	Available in 16 languages	30 minutes	Self- and observer-report. Patient, carer, and staff (non-specialist) ratings.
5. Communication Behavior in People with Dementia in Ambulant Settings (CODEMamb)	Knebel et al. (2016)	Older adults with dementia	Y	BF, A&P	Communication behaviors: verbal and non-verbal expression, social/relational communication	Available in German	3 minutes	Clinician-rated (non-specialist, trained observers). Ratings based on observations.
6. Montreal Evaluation of Communication Questionnaire for Use in Long-Term Care (MECQ-LTC)	Le Dorze et al. (2000)	Older adults in long-term care	Y	A&P, EF	Number and frequency of expressive and receptive communication acts; effort required by caregivers for efficient communication.	Available in French	30 minutes	Observer-report and clinician-rated (specialist). Ratings based on interview with caregivers and observations.
7. Communication Behavior in Dementia (CODEM)	Kuemmel et al. (2014)	Older adults with dementia	Y	BF, A&P	Communication behaviors: verbal and non-verbal expression, social/relational communication	Available in German	3 minutes	Observer-report (non-specialist, trained). Ratings based on observations.
8. Communication checklist	Dodd et al. (1990)	Older adults in long-term care	Y	BF, A&P, EF	Hearing, speech intelligibility, language, communication, communication aids	–	15 minutes	Observer-report by care staff.
9. Communication difficulty of home-bound older adult	Rippy et al. (1986)	Older adults living at home	Y	BF, EF	Verbal comprehension and expression; nurses’ comprehension of older person.	–		Clinician-rated (non-specialist). Ratings based on observations.
10. Communication plans	Généreux et al. (2004)	Older adults in long-term care	Y	BF, A&P, EF, PF	Communication abilities/strategies and communication preferences	–		Observer-report/clinician-rated (specialist)/performance based. Professional and non-professional ratings. Speech pathologist assessment. Administration of MECQ-LTC.
11. Communication Profile for the Hearing Impaired (CPHI)	Demorest and Erdman (1987)	Adults with hearing loss	N	BF, A&P, EF, PF	Communication performance, communication environment, communication strategies, and personal adjustment of hearing-impaired adults	Available in Swedish, Norwegian, Dutch	30-45 minutes	Self-report. Rating scales completed by person with hearing impairment.
12. Communication Self-Assessment Scales for Older Adults (CSOA)	Kaplan et al. (1997)	Older adults with hearing loss	Y	BF, A&P, EF, PF	Communication strategies, Communication attitudes	Available in Korean		Self-report. Rating scales completed by older adult with hearing loss.
13. Communication‐Support Needs Assessment Tool for Dementia (CoSNAT‐D)	Krein et al. (2022)	Older adults with dementia	Y	BF, A&P, EF, PF	Hearing, vision, pre-morbid language/communication difficulties, languages spoken at home, level of education, social personality, verbal expression, auditory comprehension, writing, reading, functional communication	–		Self-report, observer-report, clinician-rated (non-specialist). Likert scale ratings completed by: person living with dementia, their carer and the administering healthcare professional.
14. Everyday Digital Literacy Questionnaire for Older Adults (EDLQ)	Choi et al. (2023)	Community-dwelling older adults	Y	A&P	Digital literacy: communication using messaging, email, video calls, social media posts.	Available in Korean		Self-report. Rating scales completed by older adult.
15. Experienced Communication in Dementia (ECD-C & ECD-P)	Olthof-Nefkens et al. (2023)	Older adults with dementia and their caregivers	Y	BF, A&P, EF	Caregiver perceptions of communication and conversation challenges and psychosocial impacts. Person with dementia’s awareness and perceptions of communication and conversation challenges and psychosocial impacts.	Available in Dutch	10 minutes	Self-report and observer-report. Self-ratings and observer ratings completed by person with dementia and caregiver.
16. Hearing Attitudes to Rehabilitation Questionnaire (HARQ)	Hallam and Brooks (1996)	People with hearing loss	N	BF, A&P, EF, PF	Attitudes, beliefs, and readiness toward hearing rehabilitation and hearing aid use	Available in Persian		Self-report. Rating scale completed by person with hearing loss.
17. Hearing Handicap Inventory for the Elderly (HHIE)-short	Ventry and Weinstein (1982)	Older adults with hearing loss	Y	BF, A&P, PF	Emotional consequences of hearing impairment, social and situational effects.	Available in French, Spanish, Arabic, Chinese, Italian, Nepali	10 minutes	Self-report. Multiple choice questions completed by older adult with hearing loss.
18. Hearing Handicap Questionnaire (HHQ)	Gatehouse and Noble (2004)	Adults with hearing loss	N	BF, A&P	Impact of hearing loss on emotional wellbeing, and social and general participation.	Available in Spanish, Chinese, Indian, Portuguese, Swedish Persian, Italian, Japanese, Arabic		Self-report. Rating scale completed by person with hearing loss.
19. InterRAI Community Health Assessment (CHA)	Morris et al. (2010)	Adults in community settings	N	BF	Functional vision and hearing.	Available in French	90 minutes	Self-report and observer-reports. Reports from adults and their caregivers and/or health care providers; medical records; and social workers’ observations
20. Life-Worldly Communication Scale	Fukaya et al. (2024)	Older adults in long term care	Y	BF, A&P	Word use, conversation topics and communication of emotions. Conversation initiation, participation, maintenance, and turn-taking.	Available in Japanese		Self-report. Rating scale completed by older adult.
21. Low Vision Visual Functioning Questionnaire (LV-VFQ)-short	The National Eye Institute 25-Item Visual Function Questionnaire (VFQ-25) (2000)	Adults with vision loss	N	BF, A&P	Functional reading activities.	Available in Italian, French, German, Spanish, Turkish, Chinese, Japanese, Greek, Portuguese, Arabic, Serbian		Self-report.
22. Modified communication environment assessment and planning guide (based on Lubinski, 1995)	Hickson et al. (2005)	Older people in long term care	Y	EF	Visual, auditory, tactile, olfactory, spatial, psychosocial environments	–		Observer-rated.
23. Quantified Denver Scale of Communicative Function (QDS)-short	Tuley et al. (1990)	Adults with hearing loss	N	BF, A&P, EF	Participation restrictions, impact on family relationships	–		Self-report. Rating scales completed by adult with hearing loss.
24. Questionnaire of communication	Hickson et al. (2005)	Older people in long term care	Y	A&P, EF	Current communication interactions within long term care environment.	–		Self-report. Questionnaire completed by older adult.
25. Quick reference communication guide	Vermilya and Stevens (2012)	Adults with aphasia in inpatient rehabilitation settings	N	BF, A&P, EF	Communication practices and communication strategies	Picture based version		Observer reported by speech pathologist.
26. Self-Assessment/significant other of Communication (SAC & SOAC)	Schow and Nerbonne (1982)	Adults (including older adults) with hearing loss and their significant other.	Y	BF, A&P, EF	Participation in communication activities, attitudes toward hearing impairment.	Available in Afrikaans, Portuguese		Self-report and observer-report. Rating scales completed by adult with hearing loss and their significant other.
27. Speech, Spatial, and Qualities of Hearing Scale (SSQ)	Gatehouse and Noble (2004)	Adults with hearing loss	N	BF, A&P	Speech hearing, spatial hearing, qualities of hearing.	Available in Dutch, Korean, German, French, Persian, Russian, Danish, Polish, African, Turkish, Spanish		Self report. Rating scale completed in interview.
28. Subjective hearing capacity	Wahl et al. (2012)	Older adults with hearing and vision loss	Y	BF	Hearing capacity	Available in German		Self-report. Single item rating scale completed by older adult.
29. Subjective vision capacity	Wahl et al. (2012)	Older adults with hearing and vision loss	Y	BF	Vision capacity	Available in German		Self-report. Single item rating scale completed by older adult.

*Note*. A&P, activities and participation; BF, body functions; EF, environmental factors; ICF, International Classification of Functioning, Disability and Health; PF, personal factors; QOL, quality of life.

a Quality of life is construct not included within the ICF.

### Description of measurement instruments

#### Context

##### Scope

Included studies reported: instrument development/validation (Choi et al., 2023; Fukaya et al., 2024; Gatehouse & Noble, 2004; Knebel et al., 2016; Krein et al., 2022; Kuemmel et al., 2014; Le Dorze et al., 2000; Olthof-Nefkens et al., 2023; Reynolds et al., 2000; Schow & Nerbonne, 1982; Tuley et al., 1990; Ventry & Weinstein, 1982); intervention studies (Hickson et al., 2005, 2007; Kaplan et al., 1997; Leroi et al., 2020); comparative studies (Garstecki & Erler, 1999; Urqueta Alfaro et al., 2019; Wahl et al., 2012); observational studies (Davidson et al., 2003); and a cross-sectional study (Lee & Ha, 2024). Two publications described use in clinical settings: a clinical intervention (Généreux et al., 2004) and a case study (Vermilya & Stevens, 2012), respectively. Some measurement instruments were not explicitly used clinically in identified publications, but have the potential for clinical application (1–7, 9, 11–13, 15–19, 21, 23, 26–29). The majority of measurement instruments identified (20/29) had been validated in research studies.

##### Setting

Measurement instruments were developed and implemented in 10 different countries, including in Australia (1, 8, 13, 16, 18, 22, 24), the United States (9, 11, 12, 17, 23, 25, 26), Germany (5, 7, 28, 29), the United Kingdom (4, 21, 27), Korea (2, 14), Canada (6, 10, 19), France (3, 21), Japan (20), Cyprus (21), and the Netherlands (15). A total of 13 measurement instruments were used in care settings, including long-term care homes (6–8, 10, 20, 22, 24) and clinics (5, 28, 29), hospitals (3, 4), dementia assessment wards (4), elder hostel programs (12), communal care settings (3), and outpatient services (4). Only one measurement instrument (25) reported a picture-based accessible version. Nineteen instruments were available in multiple languages (1, 3–7, 11, 12, 14, 15, 16, 17, 19–21, 26–29).

##### Participants

The age of participants in included studies ranged from 52 to 94 years. Ten out of twenty-nine instruments were specifically developed for older adults. The target population for measurement instruments included adults with neurologically-based communication disorders (1, 2), adults with dementia (3, 5, 7, 13, 15). adults with mental illness (4), older adults in long-term care (6, 8, 10, 20, 22, 24), older adults living at home (9), adults with hearing loss (11, 12, 16, 17, 18, 23, 26, 27), adults in community settings (14, 19), adults with vision loss (19, 21), people with aphasia in inpatient rehabilitation settings (25), adults with both hearing and vision loss (28,29). The sample sizes of participants reported in publications varied considerably from 1 to 1,016 older adults.

#### Content

##### Constructs measured

Twenty-two out of twenty-nine measurement instruments contained items assessing communication activities and participation. Conversation was a key focus of measurement (3, 5–8, 11–13, 15, 16, 20, 24, 26, 27). Other measurement instruments focused on interpersonal communication (3, 5, 6, 7, 11, 12, 15–17, 23), use of communication devices (3, 12–15, 17, 21, 26, 27), daily activities/life events (5, 7, 11–13, 17, 18, 21, 23, 26), general communication (5, 7, 10, 11, 13, 15, 18, 24), listening (11, 20, 26, 27), reading (3, 13, 21), speaking (3, 6, 20, 25), writing (3, 12, 25), and non-verbal communication (3, 5, 7, 25). Body functions were frequently measured in relation to mental/emotional responses (e.g., attention; 7, 10–13, 15–18, 23, 26), hearing (4, 8, 11, 12, 16, 17, 27, 28), language comprehension (5–9, 13, 25), language expression (3, 5, 8, 9, 13), vision (4, 21, 29), cognition (8, 13), and speech (13). Several measurement instruments assessed the impact of environmental factors on communication, including support/relationships (8–13, 15, 16, 22, 23, 25, 26), attitudes (11, 12, 16, 22, 26), the natural environment (11, 22, 23, 26), services/systems/policies (8, 22, 24), and products/technology (8, 22). Six measurement instruments contained items measuring the impact of personal factors which included languages spoken at home (10, 13), personal adjustment (11) and level of education (13). Only one measurement instrument (2) measured quality of life. One measurement instrument assessed both communication support needs and preferences (10).

##### Items/response types

The number of items/questions included in each measurement instrument varied from 1 to 145. Instruments had: between 1 and 10 items (9, 10, 13, 24, 25, 27, 29, 30), 11 and 20 items (2, 5, 7, 8, 16, 18, 20, 21, 26), 21 and 30 items (4, 14, 15, 17), 31 and 40 items (1, 3, 6), 41 and 50 items (12, 22, 28), and two instruments had more than 50 items (11, 19). The response types and rating scales used in these instruments/subsets were diverse, with open-ended questions (8, 10, 25) and forced-choice questions (8, 17, 19, 22, 25, 26) being the most common. Likert scales were employed in various formats: 3-point scales (6, 12, 13, 16, 25); 4-point scales (4, 9, 15, 20); 4- to 5-point scale (21); 5-point scales (2, 11, 14, 18, 24, 27, 29, 30); 6-point scales (5, 7); 10-point scale (28); 3- to 8-point scale (3), and 6- to 8-point scale (1,19).

#### Administration

##### Mode

Administration methods included interviews (11/29), observations (8/29), and questionnaires (9/29). Two publications reported the use of pencil-and-paper based versions (Garstecki & Erler, 1999; Kaplan et al., 1997), and one was completed in-person using a tablet (Choi et al., 2023). In-person administration was the predominant mode (22/29), seven publications did not report the mode of administration. No instruments were reported to be administered online.

##### Method of report

Seventeen measurement instruments used self-report (2, 4, 11–14, 16–18, 20, 21, 23, 24, 26–29), ten used observer-report (1, 3, 5–10, 22, 25), and five used both (4, 13, 15, 19, 26). Some instruments collected data from support people (4, 6, 13, 15, 19, 26) and staff/health professionals (4, 6, 8, 13, 19, 25). Most of instruments (26/29) were administered by non-specialists. A majority of publications (22/29) did not report the duration of administration, those that did report on duration were completed within <5 minutes (5, 7), 10–15 minutes (2), 20–30 minutes (1, 4, 6), 30–45 minutes (11).

### Instrument selection

We acknowledge that there may be trade-off between selecting a comprehensive and feasible instrument for identifying older adults’ communication support needs and preferences in an aged care context. Instruments that capture rich data on real-life behaviors (e.g., Montreal Evaluation of Communication Questionnaire for Use in Long Term Care) may often require significant time and specialists to administer, which can limit their feasibility for routine use in aged care settings. On the other hand, shorter instruments that require minimal specialized training (e.g., Communication checklist) may be more suitable for everyday care but could lack strong validation, which can limit their effectiveness for guiding clinical interventions. Instruments that offer a practical compromise by balancing broad scope with ease of use may be particularly beneficial. For example, instruments that are quick to administer and suitable for use by non-specialists—while still identifying barriers and facilitators that clinical assessments may overlook—can provide valuable insights into the personal, social, and environmental factors shaping communication. These instruments help bridge the gap between clinical rigor and real-world relevance. Ultimately, instrument development and selection must reflect the realities of aged care practice—favoring inclusivity, practicality, and relevance to support equitable and effective care planning.

## Discussion

In this scoping review, we identified a total of 29 instruments used to measure older adults’ communication support needs and preferences. Identified instruments focused on the measurement of communication impairment or disability (often with reference to a single, specific health condition), with little consideration to complex or intersecting communication needs, cultural and/or linguistic background, communication preferences, or the communication environment. Most instruments measured communication from a single perspective, with only a handful of instruments evaluating communication from multiple perspectives (i.e., older person, family/carer and health professional perspectives). Although many instruments are available in multiple languages, only one was an accessible/picture-based instrument.

The majority of identified instruments were designed to measure communication in terms of discrete areas of impairment or health conditions (e.g., hearing loss or dementia), rather than communication needs relating to a particular context (i.e., residential and/or community aged care) or population (i.e., older adults). In line with the findings from a recent review by Manchha et al. (2025), which explored the intersectionality of factors affecting communication in aged care, the measurement instruments identified tended to focus on specific health conditions or communication functions, rather than capturing a broad range of factors impacting communication in care settings. Intergenerational communication differences, cultural and linguistic background, personal interests, modes of communication, and relationships with communication partners are examples of the many factors that need to be considered to understand a person’s communication needs and preferences. Many instruments measured more than one ICF domain, but few accounted for the intersection between body functions, activities and participation, and environmental and personal factors. Six of the 29 instruments measured personal factors, but only two—the Communication Checklist and the Communication-Support Needs Assessment Tool for Dementia considered whether older adults spoke a language other than English. While these tools may help identify whether individuals come from diverse cultural or linguistic backgrounds (Xiao et al., 2023), this approach is problematic, as it can imply that there are limited resources to support the language needs of these care recipients. Instead, instruments should aim to identify an older adult’s language background and examine how it shapes their communication needs. This shift would help reframe linguistic diversity as a factor influencing communication, rather than as a deficit (Manchha et al., 2025).

Likewise, there is a need to identify and document communication preferences in order to promote person-centered communication and improved care outcomes (Lombard et al., 2021). Only Communication Plans (Généreux et al., 2004) measured both communication needs and preferences and includes free text response options for recording the communication preferences of the person receiving aged care services. We did not find any instruments that identified preferences related to gender, Indigenous and/or sexual orientation. This gap limits the ability of measurement instruments to support autonomy identity expression, and inclusive care practices. The absence of such instruments can also lead to care planning failures by overlooking key aspects of a person’s identity, which are shaped by their lived experiences, communication preferences, life roles, and values. Consequently, care delivery may reinforce inequities, which can then reduce the effectiveness of support provided. Findings from this review underline the importance of developing an instrument for assessing communication support needs and preferences in the aged care context, that moves beyond discrete health conditions and body functions, to include preferences and contextual factors known to impact communication. Specifically, this review highlights the need for co-design approaches that actively engage diverse older adult populations, ensuring their voices and experiences shape the development of inclusive and effective care instruments. Without the integration of user needs and equity principles, these instruments cannot and will not support communication rights in practice.

Analysis of the evidence identified in this review suggests that many existing measurement instruments may not be feasible to implement in aged care settings. Many instruments require administration by specialists (e.g., speech pathologists, audiologists); this is a challenge given that the direct aged care workforce is predominately comprised personal care assistants and nursing professionals, and that demand for specialist clinicians far outweighs availability (Davis et al., 2024). Subsequently, no instrument is currently fit-for-purpose for broad implementation in aged care settings as profiling communication in aged care relies on general nursing or administrative staff capacity to complete assessments. This is problematic given that this same workforce has limited opportunities to receive communication-specific training (Burton et al., 2025) and/or guidance in selecting appropriate measurement instruments (Rommerskirch-Manietta et al., 2022). Thus, future research needs to focus on developing training and guidelines to support the identification of communication needs. These instruments can be potentially integrated into various aspects of care provision, such as staff training, policy accreditation, and electronic health record systems. Doing so would help prioritize communication support needs, leading to better-informed decisions and more personalized care.

Most of the identified instruments are inaccessible or not adaptable for diverse care settings. The Quick Reference Communication Guide (Vermilya & Stevens, 2012) was the only instrument to report that older adults with varying communication challenges may require communication accessible versions of measurement instruments and includes images to support written text. The lack of communication-accessible versions (i.e., easy-read versions incorporating larger font, more white space, visual aids) of measurement instruments can silence groups receiving care services and/or potentially result in a one-dimensional understanding of communication (Nocivelli et al., 2023). Future instrument development should focus not only on the content but also on the format and mode, ensuring that where possible, older adults can use accessible versions (e.g., Auslan, plain English, easy English) or versions suitable for older adults with complex health conditions (e.g., Aphasia). Although we found a majority of measurement instruments were available in multiple languages, depending on which languages were used in the adapted versions, existing instruments may not be accessible to specific language groups. We recognize that culture plays a significant role in shaping communication styles, preferences, and expectations, therefore instruments need to be tested for cultural relevance and appropriateness (Beaulieu & Jimenez‐Gomez, 2022) within the populations they aim to serve. This is challenging as it cannot be assumed that people from the same culture or generation have the same needs. It highlights the need for instruments, which are flexible, person-centered and which account for both the cultural context and individual preferences.

Facilitators for implementing profiling instruments in care contexts were also identified in this review. Although most publications did not specifically report the duration of instrument administration, we recognize that instruments that can be completed in a short amount of time are well-suited for staff in long-term care settings, where high workloads and limited time for focusing on communication must be taken into account (Forsgren & Saldert, 2022). It is acknowledged that staff may be required to rely on information from proxy assessments via family or caregivers (Milte et al., 2023). In this respect, some instruments, including the Communication‐Support Needs Assessment Tool for Dementia (Krein et al., 2022) involve a comprehensive assessment of communication based on insight from multiple perspectives (e.g., older adults, significant others, health professionals). This triangulation of perspectives is particularly beneficial as it combines the in-depth, long-standing knowledge of family and significant others with the day-to-day understanding and insights of communication partners within the care setting (e.g., nursing staff, administration staff, lifestyle staff) (Bennett et al., 2016). Future research should focus on developing instruments that accommodate multi-stakeholder and inter-professional perspectives, to ensure that measurement instruments draw on in-depth personal understanding of the person, as well as insights from the care environment.

### Strengths and limitations

To the authors’ knowledge, this is the first review to examine and describe existing measurement instruments that profile older adults’ communication support needs and preferences. The strengths of this review include the inclusion of gray literature, international studies, and examination of multiple care settings. In addition to searching academic databases, we reviewed resources published by leading industry and advocacy organizations. This enabled extension of the literature included by broadening the search to include instruments developed in practice that may be relevant. Our description of measurement instruments was limited by incomplete reporting. A significant proportion of publications did not specify the mode or duration of administration. This research was also limited by exclusion of studies in languages other than English, with 75 non-English language studies being excluded at the title-abstract stage, which may limit the variety of instruments that could support a culturally responsive approach. These limitations highlight potential opportunities for future instrument development.

## Conclusion

This scoping review examined existing observational and self-report measurement instruments for profiling communication support needs and preferences in older adults, identifying 29 instruments across 26 publications. No single instrument was suitable for comprehensive assessment of communication support needs and preferences in aged care settings. Most measurement instruments focused on a single health condition or isolated communication impairments, rather than taking a comprehensive approach to communication. Communication preferences and personal factors that influence communication, including cultural and linguistic background, were rarely considered. Furthermore, while many self-report instruments were designed for completion by older adults themselves, only one offered a communication-accessible format incorporating visual aids. A condition-agnostic measurement instrument that adopts a holistic, biopsychosocial perspective on communication, incorporates the unique environmental influences of aged care settings, draws on multiple stakeholder perspectives, and is designed to be communication-accessible for older adults with diverse communication abilities, is needed. The development of such an instrument represents a critical first step toward ensuring accurate, consistent, and timely identification of communication needs and preferences among older adults residing in or receiving aged care services. This foundational work is essential to advancing a rights-based, person-centered approach to communication for older adults in aged care contexts.

## Supplementary Material

gnag033_Supplementary_Data

## Data Availability

Full references for included measurement instruments are presented in Supplementary Materials. This review was registered on Open Science Framework (https://doi.org/10.17605/osf.io/ubk4x).
