# Supplementary material for: Identifying older adults’ communication support needs and preferences: a scoping review of measurement instruments
Source: Gerontologist. 2026 Apr 7;66(6):gnag033. doi: 10.1093/geront/gnag033 (PMC13180643; doi:10.1093/geront/gnag033)
Supplement: gnag033_Supplementary_Data [file gnag033_supplementary_data.docx]

**Identifying Older Adults’ Communication Support Needs and Preferences: A Scoping Review of Measurement Instruments**

Asmita V. MANCHHA PhD^1,2^, Bridget BURTON BSpPath^1,2^, Michelle KING PhD^1,2^, Chloe TANSWELL BSpPath^1,2^, Samantha SIYAMBALAPITIYA PhD^4^, Joanne M. WOOD PhD^5^, Louise HICKSON PhD^3,6^, Deirdre FETHERSTONHAUGH PhD^7^, Kirstine SHRUBSOLE PhD^1,2^, Geoff ARGUS^8^, Nerina SCARINCI PhD^3,6^ & Sarah J. WALLACE PhD^1,2^

^1^ Queensland Aphasia Research Centre, School of Health and Rehabilitation Sciences, The University of Queensland, Brisbane, Australia

^2^ STARS Education and Research Alliance, Surgical Treatment and Rehabilitation Service (STARS), The University of Queensland and Metro North Health

^3^ School of Health and Rehabilitation Sciences, The University of Queensland, Brisbane, Queensland, Australia

^4^ School of Health Sciences and Social Work, Griffith University, Gold Coast, Australia

^5^ Optometry and Vision Science, School of Clinical Sciences, Queensland University of Technology, Brisbane, Australia

^6^ Centre for Hearing Research (CHEAR), School of Health and Rehabilitation Sciences, The University of Queensland, Brisbane, Queensland, Australia

^7^ Australian Centre for Evidence Based Aged Care (ACEBAC), La Trobe University, Bundoora, Australia

^8^ Southern Queensland Rural Health, The University of Queensland, Toowoomba, Queensland, Australia

**ORCID**

Asmita V. MANCHHA: https://orcid.org/0000-0002-4728-0244

Bridget BURTON: https://orcid.org/0000-0003-0311-5113

Michelle KING: https://orcid.org/0000-0001-8131-4965

Chloe TANSWELL:

Samantha SIYAMBALAPITIYA: https://orcid.org/0000-0001-9310-0809

Joanne M. WOOD: https://orcid.org/0000-0002-0776-7736

Louise HICKSON: https://orcid.org/0000-0001-6832-4173

Deirdre FETHERSTONHAUGH: https://orcid.org/0000-0002-2451-3578

Kirstine SHRUBSOLE: https://orcid.org/0000-0002-7805-2447

Geoff ARGUS: https://orcid.org/0000-0001-6849-1262

Nerina SCARINCI: https://orcid.org/0000-0002-1457-6663

Sarah J. WALLACE: https://orcid.org/0000-0002-0600-9343

**Author Note**

**Correspondence**

Asmita V. Manchha, The University of Queensland

Brisbane, Queensland 4059, Australia

E-mail: a.manchha@uq.edu.au

Word count: 5252

**Supplementary Material**

Supplementary S1: Details of Database Searches for Systematic Review

Database(s): **Pubmed, PsycINFO, CINAHL, Embase**

**Pubmed**

**Set 1: Older adults and/or aged care recipient**

"older adult*"[Title/Abstract] OR "older person*"[Title/Abstract] OR "older people"[Title/Abstract] OR "elder*"[Title/Abstract] OR "aged care resident*"[Title/Abstract] OR "aged care recipient*"[Title/Abstract] OR "aged care client*"[Title/Abstract] OR "aged"[Title] OR "elder*"[Title] OR "aged care"[Title/Abstract] OR "aged-care"[Title/Abstract] OR "rest home"[Title/Abstract] OR "care home"[Title/Abstract] OR "aged care context"[Title/Abstract] OR "aged care setting"[Title/Abstract] OR "older adult care"[Title/Abstract] OR "older adult nursing"[Title/Abstract] OR "gerontolog*"[Title/Abstract] OR "geriatric*"[Title/Abstract] OR "elder care"[Title/Abstract] OR "aged care nursing"[Title/Abstract] OR "skilled nursing facilit*"[Title/Abstract] OR "care facilit*"[Title/Abstract] OR "institutionalized elder*"[Title/Abstract] OR "institutionalised elder*"[Title/Abstract] OR "gerontologic nursing"[Title/Abstract] OR "residential aged care"[Title/Abstract] OR "residential care"[Title/Abstract] OR "long term care"[Title/Abstract] OR "long-term care"[Title/Abstract] OR "nursing care facilit*"[Title/Abstract] OR "old age home"[Title/Abstract] OR "nursing home*"[Title/Abstract] OR "assisted living facilit*"[Title/Abstract] OR "home for the aged"[Title/Abstract] OR "housing for the elderly" "community aged care"[Title/Abstract] OR "retirement village"[Title/Abstract] OR "at-home care"[Title/Abstract] OR "health services for the aged"[Title/Abstract] OR "geriatric health services"[Title/Abstract] OR "health services for the elderly"[Title/Abstract] OR "geriatric health service"[Title/Abstract]

**Set 2: Tool**

"screening instrument"[Title/Abstract] OR "checklist"[Title/Abstract] OR "assessment"[Title/Abstract] OR "tool*"[Title/Abstract] OR "needs assessment"[Title/Abstract] OR "instrument"[Title/Abstract] OR "screening"[Title/Abstract] OR "evaluation"[Title/Abstract] OR "scale"[Title]

**Set 3: Communication support needs and/or preferences.**

"communication support needs"[Title/Abstract] OR "communication need*"[Title/Abstract] OR "communicat*"[Title/Abstract] OR "conversation"[Title/Abstract] OR "talk*"[Title/Abstract] OR "speech"[Title/Abstract] OR "speak*"[Title/Abstract] OR "communication barrier*"[Title/Abstract] OR "miscommunication"[Title/Abstract] OR "communication breakdown"[Title/Abstract] OR "misunderstanding"[Title/Abstract] OR "language impairment*"[Title/Abstract] OR "language disorder"[Title/Abstract] OR "Communication Aids for Disabled"[Title/Abstract] OR "communication support*"[Title/Abstract] OR "communication aid"[Title/Abstract] OR "communication management"[Title/Abstract] OR "communication strategy"[Title/Abstract] OR "communication strategies"[Title/Abstract] OR "communication disability*"[Title/Abstract] OR "communication difference*"[Title/Abstract] OR "communication impairment*"[Title/Abstract] OR "communication difficult*"[Title/Abstract] OR "communication issue*"[Title/Abstract] OR "communication disorder*"[Title/Abstract] OR "dementia" [Title/Abstract] OR "cognitive impairment" [Title/Abstract] OR "vision impairment" [Title/Abstract] OR "hearing impairment" [Title/Abstract] OR "voice impairment" [Title/Abstract] OR "speech impairment" [Title/Abstract] OR "language" [Title/Abstract] OR "neuropsychiatric" [Title/Abstract] OR "hearing loss" [Title/Abstract] OR "visually impaired persons" [Title/Abstract] OR "mental health" [Title/Abstract] OR "blind people*"[Title/Abstract] OR "deaf people" [Title/Abstract] OR "persons with hearing impairments" [Title/Abstract] OR "culturally responsive"[Title/Abstract] OR "cultural*"[Title/Abstract] OR "ethnic*"[Title/Abstract] OR "cross cultural"[Title/Abstract] OR "minority"[Title/Abstract] OR "racial"[Title/Abstract] OR "linguistically diverse"[Title/Abstract] OR "culturally diverse"[Title/Abstract] OR "CALD"[Title/Abstract] OR "linguistic diversity"[Title/Abstract] OR "bilingual"[Title/Abstract] OR "bi lingual"[Title/Abstract] OR "bi cultural"[Title/Abstract] OR "bicultural"[Title/Abstract] OR "multilingual"[Title/Abstract] OR "multi lingual"[Title/Abstract] OR "cross cultural care"[Title/Abstract] OR "ethno specific"[Title/Abstract] OR "cultural diversity"[Title/Abstract] OR "multicultural*"[Title/Abstract] OR "multi cultural*"[Title/Abstract] OR "immigrant"[Title/Abstract] OR "migrant"[Title/Abstract] OR "gender differences"[Title/Abstract] OR "digital illiteracy"[Title/Abstract] OR "limited english"[Title/Abstract] OR "english as second language"[Title/Abstract] OR "intergenerational"[Title/Abstract] OR "Vulnerable population*"[Title/Abstract] OR "minority group*"[Title/Abstract] OR "Refugee*"[Title/Abstract] OR "lgbt*"[Title/Abstract] OR "LGBT*"[Title/Abstract] OR "gay"[Title/Abstract] OR "lesbian*"[Title/Abstract] OR "bisexual*"[Title/Abstract] OR "queer"[Title/Abstract] OR "transgender"[Title/Abstract] OR "non-binary"[Title/Abstract] OR "non binary"[Title/Abstract] OR "indigenous people*"[Title/Abstract] OR "aborigin*"[Title/Abstract] OR "indigenous"[Title/Abstract] OR "torres strait*"[Title/Abstract] OR "first nation*"[Title/Abstract] OR "first people*"[Title/Abstract] OR "financially disadvantaged"[Title/Abstract] OR "remote population*"[Title/Abstract] OR "isolated communit*"[Title/Abstract] OR "veteran*"[Title/Abstract] OR (("physical environment"[Title/Abstract] OR "support person*"[Title/Abstract] OR "communication style"[Title/Abstract] OR "relationships"[Title/Abstract] OR "communication device*"[Title/Abstract] OR "communication aid*"[Title/Abstract] OR "technology"[Title/Abstract] OR "health service*"[Title/Abstract] OR "work design"[Title/Abstract] OR "staff*"[Title/Abstract] OR "built environment"[Title/Abstract] OR "social environment"[Title/Abstract] OR "environment design"[Title/Abstract]) AND ("aged care"[Title/Abstract] OR "aged-care"[Title/Abstract] OR "rest home"[Title/Abstract] OR "care home"[Title/Abstract] OR "aged care context"[Title/Abstract] OR "aged care setting"[Title/Abstract] OR "older adult care"[Title/Abstract] OR "older adult nursing"[Title/Abstract] OR "gerontolog*"[Title/Abstract] OR "geriatric*"[Title/Abstract] OR "elder care"[Title/Abstract] OR "aged care nursing"[Title/Abstract] OR "skilled nursing facilit*"[Title/Abstract] OR "care facilit*"[Title/Abstract] OR "institutionalized elder*"[Title/Abstract] OR "institutionalised elder*"[Title/Abstract] OR "gerontologic nursing"[Title/Abstract] OR "residential aged care"[Title/Abstract] OR "residential care"[Title/Abstract] OR "long term care"[Title/Abstract] OR "long-term care"[Title/Abstract] OR "nursing care facilit*"[Title/Abstract] OR "old age home"[Title/Abstract] OR "nursing home*"[Title/Abstract] OR "assisted living facilit*"[Title/Abstract] OR "home for the aged"[Title/Abstract] OR "housing for the elderly" "community aged care"[Title/Abstract] OR "retirement village"[Title/Abstract] OR "at-home care"[Title/Abstract] OR "health services for the aged"[Title/Abstract] OR "geriatric health services"[Title/Abstract] OR "health services for the elderly"[Title/Abstract] OR "geriatric health service"[Title/Abstract]))

**PsycINFO**

**Set 1: Older adults and/or aged care recipient**

TI ( “older adult*” OR “older person*” OR “older people” OR “elder*” OR “aged care resident*” OR “aged care recipient*” OR “aged care client*” OR “aged” OR “elder*”) OR AB ( “older adult*” OR “older person*” OR “older people” OR “elder*” OR “aged care resident*” OR “aged care recipient*” OR “aged care client*” )

OR TI ( “aged care” OR “aged-care” OR “rest home” OR “care home” OR “aged care context” OR “aged care setting” OR “older adult care” OR “older adult nursing” OR “gerontolog*” OR “geriatric*” OR “elder care” OR "aged care nursing" OR “skilled nursing facilit*” OR “care facilit*” OR “institutionalized elder*” OR “institutionalised elder*” OR "gerontologic nursing" OR “residential aged care” OR “residential care” OR “long term care” OR “long-term care” OR “nursing care facilit*” OR “old age home” OR “nursing home*” OR “assisted living facilit*” OR “home for the aged” OR “housing for the elderly” “community aged care” OR “retirement village” OR “at-home care” OR “health services for the aged” OR “geriatric health services” OR “health services for the elderly” OR “geriatric health service” ) OR AB ( “aged care” OR “aged-care” OR “rest home” OR “care home” OR “aged care context” OR “aged care setting” OR “older adult care” OR “older adult nursing” OR “gerontolog*” OR “geriatric*” OR “elder care” OR "aged care nursing" OR “skilled nursing facilit*” OR “care facilit*” OR “institutionalized elder*” OR “institutionalised elder*” OR "gerontologic nursing" OR “residential aged care” OR “residential care” OR “long term care” OR “long-term care” OR “nursing care facilit*” OR “old age home” OR “nursing home*” OR “assisted living facilit*” OR “home for the aged” OR “housing for the elderly” “community aged care” OR “retirement village” OR “at-home care” OR “health services for the aged” OR “geriatric health services” OR “health services for the elderly” OR “geriatric health service” )

**Set 2: Tool**

TI (“screening instrument” OR "checklist” OR “assessment” OR “scale” OR “tool*” OR “needs assessment” OR “instrument” OR “screening” OR “evaluation” ) OR AB ( “screening instrument” OR "checklist” OR “assessment” OR “tool*” OR “needs assessment” OR “instrument” OR “screening” OR “evaluation” )

**Set 3: Communication support needs and/or preferences.**

TI ( “communication support needs” OR “communication need*” OR “communicat*” OR “conversation” OR “talk*” OR “speech” OR “speak*” OR “communication barrier*” OR “miscommunication” OR “communication breakdown” OR “misunderstanding” OR “language impairment*” OR “language disorder” OR "Communication Aids for Disabled" OR “communication support*” OR “communication aid” OR “communication management” OR “communication strategy” OR “communication strategies” OR “communication disability*” OR “communication difference*” OR “communication impairment*” OR “communication difficult*” OR “communication issue*” OR “communication disorder*” ) OR AB ( “communication support needs” OR “communication need*” OR “communicat*” OR “conversation” OR “talk*” OR “speech” OR “speak*” OR “communication barrier*” OR “miscommunication” OR “communication breakdown” OR “misunderstanding” OR “language impairment*” OR “language disorder” OR "Communication Aids for Disabled" OR “communication support*” OR “communication aid” OR “communication management” OR “communication strategy” OR “communication strategies” OR “communication disability*” OR “communication difference*” OR “communication impairment*” OR “communication difficult*” OR “communication issue*” OR “communication disorder*” ) OR TI ( “dementia” OR “cognitive impairment” OR “vision impairment” OR “hearing impairment” OR “voice impairment” OR “speech impairment” OR “language” OR “neuropsychiatric” OR “hearing loss” OR “visually impaired persons” OR “mental health” OR “blind people*” OR “deaf people” OR “persons with hearing impairments” OR TI “culturally responsive” OR “cultural*” OR “ethnic*” OR “cross cultural” OR “minority” OR “racial” OR “linguistically diverse” OR “culturally diverse” OR “CALD” OR “linguistic diversity” OR “bilingual” OR “bi lingual” OR “bi cultural” OR “bicultural” OR “multilingual” OR “multi lingual” OR “cross cultural care” OR “ethno specific” OR “cultural diversity” OR “multicultural*” OR “multi cultural*” OR “immigrant” OR “migrant” OR “gender differences” OR “digital illiteracy” OR "limited english" OR "english as second language" OR "intergenerational" OR "Vulnerable population*" OR "minority group*" OR "Refugee*" OR “lgbt*” OR “LGBT*” OR “gay” OR “lesbian*” OR “bisexual*” OR “queer” OR “transgender” OR “non-binary” OR “non binary” OR "indigenous people*" OR “aborigin*” OR “indigenous” OR “torres strait*” OR “first nation*” OR “first people*” OR “financially disadvantaged” OR “remote population*” OR “isolated communit*” OR “veteran*” ) OR AB ( “dementia” OR “cognitive impairment” OR “vision impairment” OR “hearing impairment” OR “voice impairment” OR “speech impairment” OR “language” OR “neuropsychiatric” OR “hearing loss” OR “visually impaired persons” OR “mental health” OR “blind people*” OR “deaf people” OR “persons with hearing impairments”) OR AB (“culturally responsive” OR “cultural*” OR “ethnic*” OR “cross cultural” OR “minority” OR “racial” OR “linguistically diverse” OR “culturally diverse” OR “CALD” OR “linguistic diversity” OR “bilingual” OR “bi lingual” OR “bi cultural” OR “bicultural” OR “multilingual” OR “multi lingual” OR “cross cultural care” OR “ethno specific” OR “cultural diversity” OR “multicultural*” OR “multi cultural*” OR “immigrant” OR “migrant” OR “gender differences” OR “digital illiteracy” OR "limited english" OR "english as second language" OR "intergenerational" OR "Vulnerable population*" OR "minority group*" OR "Refugee*" OR “lgbt*” OR “LGBT*” OR “gay” OR “lesbian*” OR “bisexual*” OR “queer” OR “transgender” OR “non-binary” OR “non binary” OR "indigenous people*" OR “aborigin*” OR “indigenous” OR “torres strait*” OR “first nation*” OR “first people*” OR “financially disadvantaged” OR “remote population*” OR “isolated communit*” OR “veteran*” ) OR TI ( “physical environment” OR “support person*” OR “communication style” OR “relationships” OR “communication device*” OR “communication aid*” OR “technology” OR “health service*” OR “work design” OR “staff*” OR "built environment" OR "social environment" OR "environment design" ) OR AB ( “physical environment” OR “support person*” OR “communication style” OR “relationships” OR “communication device*” OR “communication aid*” OR “technology” OR “health service*” OR “work design” OR “staff*” OR "built environment" OR "social environment" OR "environment design" ) AND TI ( “aged care” OR “aged-care” OR “rest home” OR “care home” OR “aged care context” OR “aged care setting” OR “older adult care” OR “older adult nursing” OR “gerontolog*” OR “geriatric*” OR “elder care” OR "aged care nursing" OR “skilled nursing facilit*” OR “care facilit*” OR “institutionalized elder*” OR “institutionalised elder*” OR "gerontologic nursing" OR “residential aged care” OR “residential care” OR “long term care” OR “long-term care” OR “nursing care facilit*” OR “old age home” OR “nursing home*” OR “assisted living facilit*” OR “home for the aged” OR “housing for the elderly” “community aged care” OR “retirement village” OR “at-home care” OR “health services for the aged” OR “geriatric health services” OR “health services for the elderly” OR “geriatric health service” ) OR AB ( “aged care” OR “aged-care” OR “rest home” OR “care home” OR “aged care context” OR “aged care setting” OR “older adult care” OR “older adult nursing” OR “gerontolog*” OR “geriatric*” OR “elder care” OR "aged care nursing" OR “skilled nursing facilit*” OR “care facilit*” OR “institutionalized elder*” OR “institutionalised elder*” OR "gerontologic nursing" OR “residential aged care” OR “residential care” OR “long term care” OR “long-term care” OR “nursing care facilit*” OR “old age home” OR “nursing home*” OR “assisted living facilit*” OR “home for the aged” OR “housing for the elderly” “community aged care” OR “retirement village” OR “at-home care” OR “health services for the aged” OR “geriatric health services” OR “health services for the elderly” OR “geriatric health service” )

**CINAHL**

**Set 1: Older adults and/or aged care recipient**

TI ( “older adult*” OR “older person*” OR “older people” OR “elder*” OR “aged care resident*” OR “aged care recipient*” OR “aged care client*” OR “aged” OR “elder*” OR TI “aged care” OR “aged-care” OR “rest home” OR “care home” OR “aged care context” OR “aged care setting” OR “older adult care” OR “older adult nursing” OR “gerontolog*” OR “geriatric*” OR “elder care” OR "aged care nursing" OR “skilled nursing facilit*” OR “care facilit*” OR “institutionalized elder*” OR “institutionalised elder*” OR "gerontologic nursing" OR “residential aged care” OR “residential care” OR “long term care” OR “long-term care” OR “nursing care facilit*” OR “old age home” OR “nursing home*” OR “assisted living facilit*” OR “home for the aged” OR “housing for the elderly” “community aged care” OR “retirement village” OR “at-home care” OR “health services for the aged” OR “geriatric health services” OR “health services for the elderly” OR “geriatric health service” ) OR AB ( “older adult*” OR “older person*” OR “older people” OR “elder*” OR “aged care resident*” OR “aged care recipient*” OR “aged care client*” OR aged care” OR “aged-care” OR “rest home” OR “care home” OR “aged care context” OR “aged care setting” OR “older adult care” OR “older adult nursing” OR “gerontolog*” OR “geriatric*” OR “elder care” OR "aged care nursing" OR “skilled nursing facilit*” OR “care facilit*” OR “institutionalized elder*” OR “institutionalised elder*” OR "gerontologic nursing" OR “residential aged care” OR “residential care” OR “long term care” OR “long-term care” OR “nursing care facilit*” OR “old age home” OR “nursing home*” OR “assisted living facilit*” OR “home for the aged” OR “housing for the elderly” “community aged care” OR “retirement village” OR “at-home care” OR “health services for the aged” OR “geriatric health services” OR “health services for the elderly” OR “geriatric health service” )

**Set 2: Tool**

TI (“screening instrument” OR "checklist” OR “assessment” OR “scale” OR “tool*” OR “needs assessment” OR “instrument” OR “screening” OR “evaluation”) OR AB (“screening instrument” OR "checklist” OR “assessment” OR “tool*” OR “needs assessment” OR “instrument” OR “screening” OR “evaluation”)

**Set 3: Communication support needs and/or preferences.**

TI ( “communication support needs” OR “communication need*” OR “communicat*” OR “conversation” OR “talk*” OR “speech” OR “speak*” OR “communication barrier*” OR “miscommunication” OR “communication breakdown” OR “misunderstanding” OR “language impairment*” OR “language disorder” OR "Communication Aids for Disabled" OR “communication support*” OR “communication aid” OR “communication management” OR “communication strategy” OR “communication strategies” OR “communication disability*” OR “communication difference*” OR “communication impairment*” OR “communication difficult*” OR “communication issue*” OR “communication disorder*” OR “dementia” OR “cognitive impairment” OR “vision impairment” OR “hearing impairment” OR “voice impairment” OR “speech impairment” OR “language” OR “neuropsychiatric” OR “hearing loss” OR “visually impaired persons” OR “mental health” OR “blind people*” OR “deaf people” OR “persons with hearing impairments” ) OR AB ( “dementia” OR “cognitive impairment” OR “vision impairment” OR “hearing impairment” OR “voice impairment” OR “speech impairment” OR “language” OR “neuropsychiatric” OR “hearing loss” OR “visually impaired persons” OR “mental health” OR “blind people*” OR “deaf people” OR “persons with hearing impairments” OR “culturally responsive” OR “cultural*” OR “ethnic*” OR “cross cultural” OR “minority” OR “racial” OR “linguistically diverse” OR “culturally diverse” OR “CALD” OR “linguistic diversity” OR “bilingual” OR “bi lingual” OR “bi cultural” OR “bicultural” OR “multilingual” OR “multi lingual” OR “cross cultural care” OR “ethno specific” OR “cultural diversity” OR “multicultural*” OR “multi cultural*” OR “immigrant” OR “migrant” OR “gender differences” OR “digital illiteracy” OR "limited english" OR "english as second language" OR "intergenerational" OR "Vulnerable population*" OR "minority group*" OR "Refugee*" OR “lgbt*” OR “LGBT*” OR “gay” OR “lesbian*” OR “bisexual*” OR “queer” OR “transgender” OR “non-binary” OR “non binary” OR "indigenous people*" OR “aborigin*” OR “indigenous” OR “torres strait*” OR “first nation*” OR “first people*” OR “financially disadvantaged” OR “remote population*” OR “isolated communit*” OR “veteran*” ) OR AB ( “communication support needs” OR “communication need*” OR “communicat*” OR “conversation” OR “talk*” OR “speech” OR “speak*” OR “communication barrier*” OR “miscommunication” OR “communication breakdown” OR “misunderstanding” OR “language impairment*” OR “language disorder” OR "Communication Aids for Disabled" OR “communication support*” OR “communication aid” OR “communication management” OR “communication strategy” OR “communication strategies” OR “communication disability*” OR “communication difference*” OR “communication impairment*” OR “communication difficult*” OR “communication issue*” OR “communication disorder*” OR “culturally responsive” OR “cultural*” OR “ethnic*” OR “cross cultural” OR “minority” OR “racial” OR “linguistically diverse” OR “culturally diverse” OR “CALD” OR “linguistic diversity” OR “bilingual” OR “bi lingual” OR “bi cultural” OR “bicultural” OR “multilingual” OR “multi lingual” OR “cross cultural care” OR “ethno specific” OR “cultural diversity” OR “multicultural*” OR “multi cultural*” OR “immigrant” OR “migrant” OR “gender differences” OR “digital illiteracy” OR "limited english" OR "english as second language" OR "intergenerational" OR "Vulnerable population*" OR "minority group*" OR "Refugee*" OR “lgbt*” OR “LGBT*” OR “gay” OR “lesbian*” OR “bisexual*” OR “queer” OR “transgender” OR “non-binary” OR “non binary” OR "indigenous people*" OR “aborigin*” OR “indigenous” OR “torres strait*” OR “first nation*” OR “first people*” OR “financially disadvantaged” OR “remote population*” OR “isolated communit*” OR “veteran*”) OR TI ( “physical environment” OR “support person*” OR “communication style” OR “relationships” OR “communication device*” OR “communication aid*” OR “technology” OR “health service*” OR “work design” OR “staff*” OR "built environment" OR "social environment" OR "environment design" ) OR AB ( “physical environment” OR “support person*” OR “communication style” OR “relationships” OR “communication device*” OR “communication aid*” OR “technology” OR “health service*” OR “work design” OR “staff*” OR "built environment" OR "social environment" OR "environment design" ) AND TI ( “aged care” OR “aged-care” OR “rest home” OR “care home” OR “aged care context” OR “aged care setting” OR “older adult care” OR “older adult nursing” OR “gerontolog*” OR “geriatric*” OR “elder care” OR "aged care nursing" OR “skilled nursing facilit*” OR “care facilit*” OR “institutionalized elder*” OR “institutionalised elder*” OR "gerontologic nursing" OR “residential aged care” OR “residential care” OR “long term care” OR “long-term care” OR “nursing care facilit*” OR “old age home” OR “nursing home*” OR “assisted living facilit*” OR “home for the aged” OR “housing for the elderly” “community aged care” OR “retirement village” OR “at-home care” OR “health services for the aged” OR “geriatric health services” OR “health services for the elderly” OR “geriatric health service” ) OR AB ( “aged care” OR “aged-care” OR “rest home” OR “care home” OR “aged care context” OR “aged care setting” OR “older adult care” OR “older adult nursing” OR “gerontolog*” OR “geriatric*” OR “elder care” OR "aged care nursing" OR “skilled nursing facilit*” OR “care facilit*” OR “institutionalized elder*” OR “institutionalised elder*” OR "gerontologic nursing" OR “residential aged care” OR “residential care” OR “long term care” OR “long-term care” OR “nursing care facilit*” OR “old age home” OR “nursing home*” OR “assisted living facilit*” OR “home for the aged” OR “housing for the elderly” “community aged care” OR “retirement village” OR “at-home care” OR “health services for the aged” OR “geriatric health services” OR “health services for the elderly” OR “geriatric health service” )

**Embase**

**Set 1: Older adults and/or aged care recipient**

**'aged'**:ti AND **'older adult*'**:ab,ti OR **'older person*'**:ab,ti OR **'older people'**:ab,ti OR **'elder*'**:ab,ti OR **'aged care resident*'**:ab,ti OR **'aged care recipient*'**:ab,ti OR **'aged care client*'**:ab,ti OR **'aged care'**:ab,ti OR **'aged-care'**:ab,ti OR **'rest home'**:ab,ti OR **'care home'**:ab,ti OR **'aged care context'**:ab,ti OR **'aged care setting'**:ab,ti OR **'older adult care'**:ab,ti OR **'older adult nursing'**:ab,ti OR **'gerontolog*'**:ab,ti OR **'geriatric*'**:ab,ti OR **'elder care'**:ab,ti OR **'aged care nursing'**:ab,ti OR **'skilled nursing facilit*'**:ab,ti OR **'care facilit*'**:ab,ti OR **'institutionalized elder*'**:ab,ti OR **'institutionalised elder*'**:ab,ti OR **'gerontologic nursing'**:ab,ti OR **'residential aged care'**:ab,ti OR **'residential care'**:ab,ti OR **'long term care'**:ab,ti OR **'long-term care'**:ab,ti OR **'nursing care facilit*'**:ab,ti OR **'old age home'**:ab,ti OR **'nursing home*'**:ab,ti OR **'assisted living facilit*'**:ab,ti OR **'home for the aged'**:ab,ti OR **'housing for the elderly'**:ab,ti OR **'community aged care'**:ab,ti OR **'retirement village'**:ab,ti OR **'at-home care'**:ab,ti OR **'health services for the aged'**:ab,ti OR **'geriatric health services'**:ab,ti OR **'health services for the elderly'**:ab,ti OR **'geriatric health service'**:ab,ti

**Set 2: Tool**

**'screening instrument'**:ab,ti OR **'checklist'**:ab,ti OR **'assessment'**:ab,ti OR **'tool*'**:ab,ti OR **'needs assessment'**:ab,ti OR **'instrument'**:ab,ti OR **'screening'**:ab,ti OR **'evaluation'**:ab,ti OR **'scale'**:ti

**Set 3: Communication support needs and/or preferences.**

**'communication support needs'**:ab,ti OR **'communication need*'**:ab,ti OR **'communicat*'**:ab,ti OR **'conversation'**:ab,ti OR **'talk*'**:ab,ti OR **'speech'**:ab,ti OR **'speak*'**:ab,ti OR **'communication barrier*'**:ab,ti OR **'miscommunication'**:ab,ti OR **'communication breakdown'**:ab,ti OR **'misunderstanding'**:ab,ti OR **'language impairment*'**:ab,ti OR **'language disorder'**:ab,ti OR **'communication aids for disabled'**:ab,ti OR **'communication support*'**:ab,ti OR **'communication aid'**:ab,ti OR **'communication management'**:ab,ti OR **'communication strategy'**:ab,ti OR **'communication strategies'**:ab,ti OR **'communication disability*'**:ab,ti OR **'communication difference*'**:ab,ti OR **'communication impairment*'**:ab,ti OR **'communication difficult*'**:ab,ti OR **'communication issue*'**:ab,ti OR **'communication disorder*'**:ab,ti OR **'dementia'**:ab,ti OR **'cognitive impairment'**:ab,ti OR **'vision impairment'**:ab,ti OR **'hearing impairment'**:ab,ti OR **'voice impairment'**:ab,ti OR **'speech impairment'**:ab,ti OR **'language'**:ab,ti OR **'neuropsychiatric'**:ab,ti OR **'hearing loss'**:ab,ti OR **'visually impaired persons'**:ab,ti OR **'mental health'**:ab,ti OR **'blind people*'**:ab,ti OR **'deaf people'**:ab,ti OR **'persons with hearing impairments'**:ab,ti OR **'culturally responsive'**:ab,ti OR **'cultural*'**:ab,ti OR **'ethnic*'**:ab,ti OR **'cross cultural'**:ab,ti OR **'minority'**:ab,ti OR **'racial'**:ab,ti OR **'linguistically diverse'**:ab,ti OR **'culturally diverse'**:ab,ti OR **'cald'**:ab,ti OR **'linguistic diversity'**:ab,ti OR **'bilingual'**:ab,ti OR **'bi lingual'**:ab,ti OR **'bi cultural'**:ab,ti OR **'bicultural'**:ab,ti OR **'multilingual'**:ab,ti OR **'multi lingual'**:ab,ti OR **'cross cultural care'**:ab,ti OR **'ethno specific'**:ab,ti OR **'cultural diversity'**:ab,ti OR **'multicultural*'**:ab,ti OR **'multi cultural*'**:ab,ti OR **'immigrant'**:ab,ti OR **'migrant'**:ab,ti OR **'gender differences'**:ab,ti OR **'digital illiteracy'**:ab,ti OR **'limited english'**:ab,ti OR **'english as second language'**:ab,ti OR **'intergenerational'**:ab,ti OR **'vulnerable population*'**:ab,ti OR **'minority group*'**:ab,ti OR **'refugee*'**:ab,ti OR **'lgbt*'**:ab,ti OR **'gay'**:ab,ti OR **'lesbian*'**:ab,ti OR **'bisexual*'**:ab,ti OR **'queer'**:ab,ti OR **'transgender'**:ab,ti OR **'non-binary'**:ab,ti OR **'non binary'**:ab,ti OR **'indigenous people*'**:ab,ti OR **'aborigin*'**:ab,ti OR **'indigenous'**:ab,ti OR **'torres strait*'**:ab,ti OR **'first nation*'**:ab,ti OR **'first people*'**:ab,ti OR **'financially disadvantaged'**:ab,ti OR **'remote population*'**:ab,ti OR **'isolated communit*'**:ab,ti OR **'veteran*'**:ab,ti)

OR (**'physical environment'**:ab,ti OR **'support person*'**:ab,ti OR **'communication style'**:ab,ti OR **'relationships'**:ab,ti OR **'communication device*'**:ab,ti OR **'communication aid*'**:ab,ti OR **'technology'**:ab,ti OR **'health service*'**:ab,ti OR **'work design'**:ab,ti OR **'staff*'**:ab,ti OR **'built environment'**:ab,ti OR **'social environment'**:ab,ti OR **'environment design'**:ab,ti) AND ((**'aged care'**:ab,ti OR **'aged-care'**:ab,ti OR **'rest home'**:ab,ti OR **'care home'**:ab,ti OR **'aged care context'**:ab,ti OR **'aged care setting'**:ab,ti OR **'older adult care'**:ab,ti OR **'older adult nursing'**:ab,ti OR **'gerontolog*'**:ab,ti OR **'geriatric*'**:ab,ti OR **'elder care'**:ab,ti OR **'aged care nursing'**:ab,ti OR **'skilled nursing facilit*'**:ab,ti OR **'care facilit*'**:ab,ti OR **'institutionalized elder*'**:ab,ti OR **'institutionalised elder*'**:ab,ti OR **'gerontologic nursing'**:ab,ti OR **'residential aged care'**:ab,ti OR **'residential care'**:ab,ti OR **'long term care'**:ab,ti OR **'long-term care'**:ab,ti OR **'nursing care facilit*'**:ab,ti OR **'old age home'**:ab,ti OR **'nursing home*'**:ab,ti OR **'assisted living facilit*'**:ab,ti OR **'home for the aged'**:ab,ti OR **'housing for the elderly'**:ab,ti) AND **'community aged care'**:ab,ti OR **'retirement village'**:ab,ti OR **'at-home care'**:ab,ti OR **'health services for the aged'**:ab,ti OR **'geriatric health services'**:ab,ti OR **'health services for the elderly'**:ab,ti OR **'geriatric health service'**:ab,ti)

Reference lists of review papers identified in the search were also checked for any relevant publications that may have been missed in previous searches. *More information available on request.*

1. Grey literature via Advanced Google Search

**Round 1: *383 results screened- 2 met eligibility criteria***

All of these words: “tool” “screening instrument” “test” “scale” "checklist” "self-report" “assessment” “needs Assessment” “instrument” “screening” “evaluation” “profile” “communication needs”

This exact word or phrase: “Older adults” “Aged care” “Older people”

**Round 2: *25,600 results screened 15 pages- 3 met eligibility criteria***

This exact word or phrase: " communication checklist”

Not: child

**Round 3: *7 results- 0 met eligibility criteria***

This exact word or phrase: "aged care" "care" "communication profile" "communication checklist"

# **Supplementary S2:** Context of Included Instruments

^#^Information about this tool is derived from a secondary source, as the original source did not contain a study addressing the communication support needs and preferences of older adults. See S4 for secondary source.

|  | **Tool** | **Citation of tool** | **Intended scope of use** | **Participants** | **Study design** | **Study sample** | **Country** | **Validation** | **Versions** | **Publicly available** |
| --- | --- | --- | --- | --- | --- | --- | --- | --- | --- | --- |
|  | ASHA Functional Assessment of Communication Skills for Adults | Frattali, C.M., Thompson, C.K., Holland, A.L., Wohl, C.B., Wenck, C.J., Slater, S.C. & Paul, D. (2017) *American Speech-Language-Hearing Association Functional Assessment of Communication Skills for Adults (ASHA FACS).* https://apps.asha.org/eweb/OLSDynamicPage.aspx?Webcode=olsdetails&title=American+Speech-Language-Hearing+Association+Functional+Assessment+of+Communication+Skills+for+Adults+(ASHA+FACS) | Research & Clinical practice | Adults with neurologically-based communication disorders^#^ | Naturalistic observational study^#^ | 30 older adults aged 63-80, 8 females, 7 males^#^ | Australia | Yes | Available in English, Chinese, Italian, Portuguese | Fee/  licence |
|  | ASHA Quality of Communication Life Scale | Paul, D.R, Frattali, C.M., Holland, A.L., Thompson, C.K., Caperton, C.J., Slater, S.C. (2004) *Quality of Communication Life Scale (ASHA QCL)* https://apps.asha.org/eweb/OLSDynamicPage.aspx?Webcode=olsdetails&title=Quality+of+Communication+Life+Scale+(ASHA+QCL) | Research & Clinical practice | Adults with neurologically-based communication disorders^#^ | Cross-sectional study^#^ | 171 older adults aged 60 years or older, (82 males, 121 females) with sensorineural hearing loss^#^ | Korea | Yes | - | Fee/  licence |
|  | Behavioural Assessment Scale (BAS)- communication subscale | Ritchie, K., & Ledesert, B. (1991). The measurement of incapacity in the severely demented elderly: The validation of a behavioural assessment scale. *International Journal of Geriatric Psychiatry*, *6*(4), 217–226. https://doi.org/10.1002/gps.930060406 | Clinical practice | Older adults with dementia | Instrument development study | 322 older adults over 60 years of age (264 Females 58 Males), with a diagnosis of dementia | France | Yes | Available in French | Yes |
|  | Camberwell Assessment of Need for the Elderly (CANE) questionnaire | Reynolds, T., Thornicroft, G., Abas, M., Woods, B., Hoe, J., Leese, M., & Orrell, M. (2000). Camberwell Assessment of Need for the Elderly (CANE): Development, validity and reliability. British Journal of Psychiatry, 176(5), 444–452. https://doi.org/10.1192/bjp.176.5.444 | Research | Older adults with mental illness | Instrument development study | 55 older adults aged 59-97yrs, 29 females, 26 males | UK | Yes | Available in 16 languages | Yes |
|  | Communication Behavior in People with Dementia in Ambulant Settings (CODEMamb) | Knebel, M., Haberstroh, J., Kümmel, A., Pantel, J., & Schröder, J. (2016). CODEM amb - an observational communication behavior assessment tool for use in ambulatory dementia care. *Aging & Mental Health*, *20*(12), 1286–1296. https://doi.org/10.1080/13607863.2015.1075959 | Research | Older adults with dementia | Instrument development study | 170 older adults aged 52-88 years, (85 males, 85 females) | Germany | Yes | Available in German | Yes |
|  | Montreal Evaluation of Communication Questionnaire for Use in Long Term Care (MECQ-LTC) | Le Dorze, G., Julien, M., Genereux, S., Larfeuil, C., Navennec, C., Laporte, D., & Champagne, C. (2000). The development of a procedure for the evaluation of communication occurring between residents in long-term care and their caregivers. *Aphasiology*, *14*(1), 17-51. https://doi.org/10.1080/026870300401586 | Research | Older adults in long-term care | Instrument development study | Residents | Canada | No | Available in French | Yes |
|  | Communication Behaviour in Dementia (CODEM) | Kuemmel, A., Haberstroh, J., & Pantel, J. (2014). CODEM Instrument: Developing a Tool to Assess Communication Behaviour in Dementia. GeroPsych, 27(1), 23–31. https://doi.org/10.1024/1662-9647/a000100 | Research | Older adults with dementia | Instrument development study | 60 older adults, 80% female and 20% male, living with dementia | Germany | Yes | Available in German | Yes |
|  | Communication checklist | Dodd, B., Worrall, L., & Hickson, L. (1990). *Communication: a guide for residential care staff*. A.G.P.S. | Research | Older adults in long-term care | N/A | N/A | Australia | No | - | Yes |
|  | Communication difficulty of home-bound older adult | Rippy, J., Dancer, J., Pryor, R., & Stamper, J. (1986). A Field Experience: Rating the Communication Difficulty of Home-Bound Older Persons. *Home Health Care Services Quarterly*, *6*(3), 33–47. https://doi.org/10.1300/J027v06n03_04 | Clinical practice | Older adults living at home | Instrument development study | 25 older adults aged 53- 94 | USA | No | - | Yes |
|  | Communication plans | Généreux, S., Julien, M., Larfeuil, C., Lavoie, V., Soucy, O., & Le Dorze, G. (2004). Using communication plans to facilitate interactions with communication-impaired persons residing in long-term care institutions. Aphasiology, 18(12), 1161–1175. https://doi.org/10.1080/02687030444000507 | Clinical practice | Older adults in long-term care | Intervention | 10 residents aged 63-95 yrs (8 females, 2 males) | Canada | No | - | Yes |
|  | Communication Profile for the Hearing Impaired (CPHI) | Demorest, M. E., & Erdman, S. A. (1987). Development of the Communication Profile for the Hearing Impaired. The Journal of Speech and Hearing Disorders, 52(2), 129–143. https://doi.org/10.1044/jshd.5202.129 | Research | Hearing impaired adults^#^ | Comparative study^#^ | 301 older adults aged 65+, 159 males, 142 females^#^ | USA | Yes | Available in Swedish, Norwegian, Dutch | Yes |
|  | Communication Self-Assessment Scales for Older Adults (CSOA) | Kaplan, H., Bally, S., Brandt, F., Busacco, D., & Pray, J. (1997). Communication Scale for Older Adults (CSOA). *Journal of the American Academy of Audiology*, *8*(3), 203–217. | Research | Older adults with hearing loss | Intervention | 135 older adults aged 60-88yrs, (69 males, 66 females) | USA | Yes | Available in Korean | Yes |
|  | Communication‐support needs assessment tool for dementia (CoSNAT‐D) | Krein, L., Jeon, Y., Miller Amberber, A., & Fethney, J. (2022). Communication support needs assessment in dementia (CoSNAT‐D): An international content validation study. Health & Social Care in the Community, 30(6), e4745–e4757. https://doi.org/10.1111/hsc.13881 | Research | Older adults with dementia | Instrument development | N/A | Australia | Yes | - | Yes |
|  | Everyday Digital Literacy Questionnaire | Choi, J. Y., Choi, S., Song, K., Baek, J., Kim, H., Choi, M., Kim, Y., Chu, S. H., & Shin, J. (2023). Everyday Digital Literacy Questionnaire for Older Adults: Instrument Development and Validation Study. *JMIR. Journal of Medical Internet Research/Journal of Medical Internet Research*, *25*(5), e51616–e51616. https://doi.org/10.2196/51616 | Research | Community-dwelling older adults | Instrument development and validation study | 1016 older adults aged 60+, 530 females, 486 males | Korea | Yes | Available in Korean | Yes |
|  | Experienced Communication in Dementia (ECD-C & ECD-P) | Olthof-Nefkens, M. W. L. J., Derksen, E. W. C., Lambregts, B., de Swart, B. J. M., Nijhuis-Van der Sanden, M. W. G., & Kalf, J. G. (2023). Clinimetric Evaluation of the Experienced Communication in Dementia Questionnaire. The Gerontologist, 63(1), 40–51. https://doi.org/10.1093/geront/gnab187 | Research | Older adults with dementia and their caregivers | Instrument development study | 57 older adults (58% males, 42% females) living with dementia | The Netherlands | Yes | Available in Dutch | Yes |
|  | Hearing Attitudes to Rehabilitation Questionnaire (HARQ) | Hallam, R. S., & Brooks, D. N. (1996). Development of the Hearing Attitudes in Rehabilitation Questionnaire (HARQ). British Journal of Audiology, 30(3), 199–213. https://doi.org/10.3109/03005369609079040 | Research | Adults with hearing loss^#^ | Intervention^#^ | 178 older adults mean age 73.87 yrs (55.1% females, 44.9% males) with/without hearing aids^#^ | Australia | Yes | Available in Persian | Yes |
|  | Hearing Handicap Inventory for the Elderly (HHIE)-short | Ventry, I. M., & Weinstein, B. E. (1982). The Hearing Handicap Inventory for the Elderly: a New Tool. *Ear and Hearing*, *3*(3), 128–134. https://doi.org/10.1097/00003446-198205000-00006 | Research | Older adults with hearing loss | Instrument development study | 100 community-based older adults aged 65+ years (48 males, 52 females) | USA | Yes | Available in French, Spanish, Arabic, Chinese, Italian, Nepali | Yes |
|  | Hearing Handicap Questionnaire (HHQ) | Gatehouse, S., & Noble, W. (2004). The Speech, Spatial and Qualities of Hearing Scale (SSQ). International Journal of Audiology, 43(2), 85–99. https://doi.org/10.1080/14992020400050014 | Research | Adults with hearing loss^#^ | Intervention^#^ | 178 older adults mean age 73.87 yrs (55.1% females, 44.9% males) ^#^ with/without hearing aids | Australia | Yes | Available in Spanish, Chinese, Indian, Portuguese, Swedish Persian, Italian, Japanese, Arabic | Yes |
|  | InterRAI Community Health Assessment (CHA) | Morris, J.N, Berg, K., Bjorkgren, M, Dedercq, A., Finne-Soveri, H., Fries, B.E., Frijters, D., Gilgen, R., Gray, L., Henrard, J., Hirdes, J.P., Ljunggren, G., Nonemaker, S., Steel, K., Szczerbinska. K. (2010). InterRAI Community Health (CHA) Assessment Form and User’s Manual Version 9.1 https://catalog.interrai.org/products/978-1-936065-17-2 | Research & Clinical practice | Adults in community settings^#^ | Comparative study^#^ | 200 older adults aged 61 years, mean aged 81.3 yrs (61% females, 39% males) ^#^ | Canada | Yes | Available in French | Fee/  license |
|  | Life-Worldly Communication Scale | Fukaya, Y., Kawaguchi, M., Okabe, M., Koyama, S., & Kitamura, T. (2024). Development of the Life-Worldly Communication Scale to Improve Quality of Life: Daily Conversation as Care for Older Adults. *Health Communication*, *39*(9), 1727–1737. https://doi.org/10.1080/10410236.2023.2233180 | Research | Older adults in long term care | Instrument development study | 539 older adults aged 65–95 yrs, 355 females, 180 males | Japan | Yes | Available in Japanese | Yes |
|  | Low Vision Visual Functioning Questionnaire (LV-VFQ)-short | The National Eye Institute 25-Item Visual Function Questionnaire (VFQ-25) Version 2000 https://www.rand.org/content/dam/rand/www/external/health/surveys_tools/vfq/vfq25_manual.pdf | Research | Adults with vision loss^#^ | Intervention^#^ | 19 older adults living with dementia aged 62+, (63% males, 37% females), 42% with hearing impairment, 58% hearing and vision impairment^#^ | France, UK, Cyprus | Yes | Available in Italian, French, German, Spanish, Turkish, Chinese, Japanese, Greek, Portuguese, Arabic, Serbian | Yes |
|  | Modified communication environment assessment and planning guide | Hickson, L., Worrall, L., Wilson, J., Tilse, C., & Setterlund, D. (2005). Evaluating communication for resident participation in an aged care facility. *Advances in Speech-Language Pathology*, *7*(4), 245–257. https://doi.org/10.1080/14417040500337047 | Research | Older people in long term care | Intervention | 17 residents mean aged 89 yrs 9 months, 80% females, 20% males | Australia | No | - | Yes |
|  | Quantified Denver Scale of Communicative Function (QDS)- short | Tuley, M. R., Mulrow, C. D., Aguilar, C., & Velez, R. (1990). A Critical Reevaluation of the Quantified Denver Scale of Communication Function. *Ear and Hearing*, *11*(1), 56–61. https://doi.org/10.1097/00003446-199002000-00011 | Research | Older people in long term care | Instrument development study | 238 Older adults aged 67-77, 1% females, 99% males | USA | Yes | - | Yes |
|  | Questionnaire of communication | Hickson, L., Worrall, L., Wilson, J., Tilse, C., & Setterlund, D. (2005). Evaluating communication for resident participation in an aged care facility. *Advances in Speech-Language Pathology*, *7*(4), 245–257. https://doi.org/10.1080/14417040500337047 | Research | Older people in long term care | Intervention | 13 residents | Australia | No | - | Yes |
|  | Quick reference communication guide | Vermilya, S., & Stevens, L. C. (2012). A Guide for Enhancing Patient and Caregiver Communication. *Perspectives on Augmentative and Alternative Communication*, *21*(2), 32–36. https://doi.org/10.1044/aac21.2.32 | Clinical practice | Adults with aphasia in inpatient rehabilitation settings | Case study | 1 older adult aged 74 yrs, female | USA | No | Picture based version | Yes |
|  | Self-Assessment/significant other of Communication (SAC & SOAC) | Schow, R. L., & Nerbonne, M. A. (1982). Communication Screening Profile: Use with Elderly Clients. *Ear and Hearing*, *3*(3), 135–147. https://doi.org/10.1097/00003446-198205000-00007 | Research | Adults (including older adults) with hearing loss and their significant other. | Instrument development | N/A | USA | Yes | Available in Afrikaans, Portuguese | Yes |
|  | Speech, Spatial, and Qualities of Hearing Scale (SSQ) | Gatehouse, S., & Noble, W. (2004). The speech, spatial and qualities of hearing scale (SSQ). *International journal of audiology*, *43*(2), 85-99. | Research | Adults with hearing loss | Instrument development study | 153 older adults average age 71 yrs, (80 females, 73 males) | UK | Yes | Available in Dutch, Korean, German, French, Persian, Russian, Danish, Polish, African, Turkish, Spanish | Yes |
|  | Subjective hearing capacity | Wahl, H. W., Heyl, V., & Schilling, O. (2012). Robustness of personality and affect relations under chronic conditions: The case of age-related vision and hearing impairment. *The Journals of Gerontology. Series B, Psychological Sciences and Social Sciences*, *67*(6), 687–696. https://doi.org/10.1093/geronb/gbs002 | Research | Older adults with hearing and vision loss | Comparative study | 387 older adults aged 75-94 yrs, (194 females, 193 males), with/ without sensory impairments | Germany | No | Available in German | Yes |
|  | Subjective vision capacity | Wahl, H. W., Heyl, V., & Schilling, O. (2012). Robustness of personality and affect relations under chronic conditions: The case of age-related vision and hearing impairment. *The Journals of Gerontology. Series B, Psychological Sciences and Social Sciences*, *67*(6), 687–696. https://doi.org/10.1093/geronb/gbs002 | Research | Older adults with hearing and vision loss | Comparative study | 387 older adults aged 75-94 yrs, (194 females, 193 males), with/without sensory impairments | Germany | No | Available in German | Yes |

**Supplementary S3**: Content of Included Instruments

|  | **Tool** | **Citation of tool** | **No. of items/ questions** | **Response type/rating scale** | **Relevant communication constructs** | **Example item/question** |
| --- | --- | --- | --- | --- | --- | --- |
|  | ASHA Functional Assessment of Communication Skills for Adults | Frattali, C.M., Thompson, C.K., Holland, A.L., Wohl, C.B., Wenck, C.J., Slater, S.C. & Paul, D. (2017) *American Speech-Language-Hearing Association Functional Assessment of Communication Skills for Adults (ASHA FACS).* https://apps.asha.org/eweb/OLSDynamicPage.aspx?Webcode=olsdetails&title=American+Speech-Language-Hearing+Association+Functional+Assessment+of+Communication+Skills+for+Adults+(ASHA+FACS) | 43 | 7 point likert scale | 4 domains: social communication, communication of basic needs, reading/ writing/number concept, and daily planning | Recording the presence or absence of items in everyday communication (e.g.,greetings/intros/farewells, phone calls, reading) |
|  | ASHA Quality of Communication Life Scale | Paul, D.R, Frattali, C.M., Holland, A.L., Thompson, C.K., Caperton, C.J., Slater, S.C. (2004) *Quality of Communication Life Scale (ASHA QCL)* https://apps.asha.org/eweb/OLSDynamicPage.aspx?Webcode=olsdetails&title=Quality+of+Communication+Life+Scale+(ASHA+QCL) | 17 | 5 point Likert scale. | 4 domains: confidence and autonomy in communication, roles and self, participation in daily activities, interaction with others | It’s easy for me to communicate. (1) strongly disagree- (5) strongly agree |
|  | Behavioural Assessment Scale (BAS)- communication subscale | Ritchie, K., & Ledesert, B. (1991). The measurement of incapacity in the severely demented elderly: The validation of a behavioural assessment scale. *International Journal of Geriatric Psychiatry*, *6*(4), 217–226. https://doi.org/10.1002/gps.930060406 | 32 | 3-8 point Likert scales. Varied rating scales based on category | 5 domains: language, social integration, occupation and orientation, physical independence, mobility. Communication sub-scale measures language and social integration. | Oral comprehension: rated 5 (understands complex orders)- 0 (no reaction when spoken to) |
|  | Camberwell Assessment of Need for the Elderly (CANE) questionnaire | Reynolds, T., Thornicroft, G., Abas, M., Woods, B., Hoe, J., Leese, M., & Orrell, M. (2000). Camberwell Assessment of Need for the Elderly (CANE): Development, validity and reliability. British Journal of Psychiatry, 176(5), 444–452. https://doi.org/10.1192/bjp.176.5.444 | 24 | 4 point Likert scale. | 1 domain: eyesight/hearing | Eyesight/hearing: Rate level of need (1) no need- (3) unmet need, (4) not known |
|  | Communication Behavior in People with Dementia in Ambulant Settings (CODEMamb) | Knebel, M., Haberstroh, J., Kümmel, A., Pantel, J., & Schröder, J. (2016). CODEM amb - an observational communication behavior assessment tool for use in ambulatory dementia care. *Aging & Mental Health*, *20*(12), 1286–1296. https://doi.org/10.1080/13607863.2015.1075959 | 19 | 6 point Likert scale. | 5 domains: presentation, comprehension, remembering, new items, task and stimuli | She/he signals the need to communicate. (1) never- (6) always |
|  | Montreal Evaluation of Communication Questionnaire for Use in Long Term Care (MECQ-LTC) | Le Dorze, G., Julien, M., Genereux, S., Larfeuil, C., Navennec, C., Laporte, D., & Champagne, C. (2000). The development of a procedure for the evaluation of communication occurring between residents in long-term care and their caregivers. *Aphasiology*, *14*(1), 17-51. https://doi.org/10.1080/026870300401586 | 38 | 3 point likert scale. | 2 domains: specific life situations, generic | Statements referring to communication acts involving resident |
|  | Communication Behaviour in Dementia (CODEM) | Kuemmel, A., Haberstroh, J., & Pantel, J. (2014). CODEM Instrument: Developing a Tool to Assess Communication Behaviour in Dementia. GeroPsych, 27(1), 23–31. https://doi.org/10.1024/1662-9647/a000100 | 15 | 6 point Likert scale. | 2 domains: Verbal/content aspect, non-verbal/relationship aspect | She/he uses a sensible sentence structure. (0) never- (5) always |
|  | Communication checklist | Dodd, B., Worrall, L., & Hickson, L. (1990). *Communication: a guide for residential care staff*. A.G.P.S. | 13 | Open-ended responses.  Forced choice responses | 5 domains: hearing, speech intelligibility, language, communication, communication aids | Does this person have a obvious hearing loss? Yes, no, don’t know. |
|  | Communication difficulty of home-bound older adult | Rippy, J., Dancer, J., Pryor, R., & Stamper, J. (1986). A Field Experience: Rating the Communication Difficulty of Home-Bound Older Persons. *Home Health Care Services Quarterly*, *6*(3), 33–47. https://doi.org/10.1300/J027v06n03_04 | 3 | 4 point Likert scale. | 3 domains: patient ability to understand nurse, nurse ability to understand patient, patient ability to express ideas/feelings/needs. | Patient's ability to understand nurse. (0) person had no difficulty understanding me- (4) Person unable to understand me (100% of the time) |
|  | Communication plans | Généreux, S., Julien, M., Larfeuil, C., Lavoie, V., Soucy, O., & Le Dorze, G. (2004). Using communication plans to facilitate interactions with communication-impaired persons residing in long-term care institutions. Aphasiology, 18(12), 1161–1175. https://doi.org/10.1080/02687030444000507 | 4 | Open-ended responses | 2 domains: communication abilities/strategies and communication preferences | How to communicate with [resident's name]? |
|  | Communication Profile for the Hearing Impaired (CPHI) | Demorest, M. E., & Erdman, S. A. (1987). Development of the Communication Profile for the Hearing Impaired. The Journal of Speech and Hearing Disorders, 52(2), 129–143. https://doi.org/10.1044/jshd.5202.129 | 145 | 5 point Likert scales. Varied rating scales based on category | 4 domains: communication performance, communication environment, communication strategies, personal adjustment | Sometimes I have trouble understanding what's being said when someone speaks to me from another room. (1) Strongly Disagree to (5) Strongly agree |
|  | Communication Self-Assessment Scales for Older Adults (CSOA) | Kaplan, H., Bally, S., Brandt, F., Busacco, D., & Pray, J. (1997). Communication Scale for Older Adults (CSOA). *Journal of the American Academy of Audiology*, *8*(3), 203–217. | 41 | 3 point Likert scale. | 2 domains: Communication Strategies scale, Communication Attitudes scale | You are talking with someone you do not know well. You do not understand. You ask her to repeat. (1) almost always, (2) sometimes, (3) almost never |
|  | Communication‐support needs assessment tool for dementia (CoSNAT‐D) | Krein, L., Jeon, Y., Miller Amberber, A., & Fethney, J. (2022). Communication support needs assessment in dementia (CoSNAT‐D): An international content validation study. Health & Social Care in the Community, 30(6), e4745–e4757. https://doi.org/10.1111/hsc.13881 | 8 | 3 point Likert scale. | Pre-commencement questions. 5 domains: verbal expression, auditory comprehension, writing, reading, functional communication | Do you feel you have difficulty holding a one-on-one conversation? (1) never or rarely- (3) frequently or almost always |
|  | Everyday Digital Literacy Questionnaire | Choi, J. Y., Choi, S., Song, K., Baek, J., Kim, H., Choi, M., Kim, Y., Chu, S. H., & Shin, J. (2023). Everyday Digital Literacy Questionnaire for Older Adults: Instrument Development and Validation Study. *JMIR. Journal of Medical Internet Research/Journal of Medical Internet Research*, *25*(5), e51616–e51616. https://doi.org/10.2196/51616 | 22 | 5 point Likert scale. | 3 domains: information and communication, contents creation, management, safety and security | [I can] participate in video calls or conferences using digital devices. (1) strongly disagree- (5) strongly agree |
|  | Experienced Communication in Dementia (ECD-C & ECD-P) | Olthof-Nefkens, M. W. L. J., Derksen, E. W. C., Lambregts, B., de Swart, B. J. M., Nijhuis-Van der Sanden, M. W. G., & Kalf, J. G. (2023). Clinimetric Evaluation of the Experienced Communication in Dementia Questionnaire. The Gerontologist, 63(1), 40–51. https://doi.org/10.1093/geront/gnab187 | 24-29 | 4-point likert scales. Varied rating scales based on category | 2-3 domains: experienced communication (caregiver competence, social communication, communication difficulties in daily life, and experienced emotions during conversations). judgment of the conversation quality. caregiver’s experienced emotions regarding the communication problems | I feel nervous during a conversation/My partner feels nervous during a conversation. During every conversation–(3) every day–(2) every week–(1) almost never (0) never |
|  | Hearing Attitudes to Rehabilitation Questionnaire (HARQ) | Hallam, R. S., & Brooks, D. N. (1996). Development of the Hearing Attitudes in Rehabilitation Questionnaire (HARQ). British Journal of Audiology, 30(3), 199–213. https://doi.org/10.3109/03005369609079040 | 20 | 3 point Likert scale. | 4 domains: personal distress/inadequacy, hearing loss stigma, minimization of hearing impairment, normal hearing | My hearing loss makes me feel isolated from other people. (1) not true- (3) true |
|  | Hearing Handicap Inventory for the Elderly (HHIE)-short | Ventry, I. M., & Weinstein, B. E. (1982). The Hearing Handicap Inventory for the Elderly: a New Tool. *Ear and Hearing*, *3*(3), 128–134. https://doi.org/10.1097/00003446-198205000-00006 | 25 | Forced choice responses | 2 domains: emotional consequences of hearing impairment, social and situational effects. | Does a hearing problem cause you to use the phone less often than you would like? (4) Yes, (2) sometimes, (0) no |
|  | Hearing Handicap Questionnaire (HHQ) | Gatehouse, S., & Noble, W. (2004). The Speech, Spatial and Qualities of Hearing Scale (SSQ). International Journal of Audiology, 43(2), 85–99. https://doi.org/10.1080/14992020400050014 | 12 | 5 point Likert scale. | 3 domains: emotional distress and discomfort, social withdrawal, general restriction on participation | How often does your hearing difficulty restrict the things you do? (1) never- (5) almost always |
|  | InterRAI Community Health Assessment (CHA) | Morris, J.N, Berg, K., Bjorkgren, M, Dedercq, A., Finne-Soveri, H., Fries, B.E., Frijters, D., Gilgen, R., Gray, L., Henrard, J., Hirdes, J.P., Ljunggren, G., Nonemaker, S., Steel, K., Szczerbinska. K. (2010). InterRAI Community Health (CHA) Assessment Form and User’s Manual Version 9.1 https://catalog.interrai.org/products/978-1-936065-17-2 | 150 items | Forced choice responses. 6-8 point Likert scales | 13 domains: cognition, social functioning, functional vision, hearing, communication | [observe] ability to understand others (comprehension) (0) intact-(8) very severe impairment |
|  | Life-Worldly Communication Scale | Fukaya, Y., Kawaguchi, M., Okabe, M., Koyama, S., & Kitamura, T. (2024). Development of the Life-Worldly Communication Scale to Improve Quality of Life: Daily Conversation as Care for Older Adults. *Health Communication*, *39*(9), 1727–1737. https://doi.org/10.1080/10410236.2023.2233180 | 11 | 4 point Likert scale. | 5 domains: talk about life world topics, emotional interchange, sharing personally meaningful worlds, relationship building, autonomous construction of the conversation | [I can] talk spontaneously (0) strongly disagree- (3) strongly agree |
|  | Low Vision Visual Functioning Questionnaire (LV-VFQ)-short | The National Eye Institute 25-Item Visual Function Questionnaire (VFQ-25) Version 2000 https://www.rand.org/content/dam/rand/www/external/health/surveys_tools/vfq/vfq25_manual.pdf | 20 | 4-5 point Likert scales. Varied rating scales based on category. | 2 domains: Vision difficulties, vision communication aids | Is it difficult to read mail? (1) not difficult- (4) impossible. |
|  | Modified communication environment assessment and planning guide | Hickson, L., Worrall, L., Wilson, J., Tilse, C., & Setterlund, D. (2005). Evaluating communication for resident participation in an aged care facility. *Advances in Speech-Language Pathology*, *7*(4), 245–257. https://doi.org/10.1080/14417040500337047 | 48 | Forced choice responses | 5 domains: visual environment, auditory environment, tactile and olfactory environment, spatial, psychosocial | Can background noise be controlled during conversations? |
|  | Quantified Denver Scale of Communicative Function (QDS)- short | Tuley, M. R., Mulrow, C. D., Aguilar, C., & Velez, R. (1990). A Critical Reevaluation of the Quantified Denver Scale of Communication Function. *Ear and Hearing*, *11*(1), 56–61. https://doi.org/10.1097/00003446-199002000-00011 | 5 | 5 point likert scale. | 2 domains: participation restrictions, impact on family relationships | I am not an outgoing person because of my hearing loss. (1) strongly disagree- (5) strongly agree |
|  | Questionnaire of communication | Hickson, L., Worrall, L., Wilson, J., Tilse, C., & Setterlund, D. (2005). Evaluating communication for resident participation in an aged care facility. *Advances in Speech-Language Pathology*, *7*(4), 245–257. https://doi.org/10.1080/14417040500337047 | 6 | Open-ended questions. Forced choice responses. 3 point likert scale. | 2 domains: communication practices and communication opportunities | How would you rate your communication (quality & quantity)? (1) good, (2) ok, (3) poor |
|  | Quick reference communication guide | Vermilya, S., & Stevens, L. C. (2012). A Guide for Enhancing Patient and Caregiver Communication. *Perspectives on Augmentative and Alternative Communication*, *21*(2), 32–36. https://doi.org/10.1044/aac21.2.32 | 12 | Forced choice responses | 2 domains: communication practices and communication strategies | I communicate best by. (gestures, head nods/shakes, speaking, writing) (Tick the box) |
|  | Self-Assessment/significant other of Communication (SAC & SOAC) | Schow, R. L., & Nerbonne, M. A. (1982). Communication Screening Profile: Use with Elderly Clients. *Ear and Hearing*, *3*(3), 135–147. https://doi.org/10.1097/00003446-198205000-00007 | 10 | 5 point Likert scale. | 3 domains: various communication situations, feelings about communication, other people | Do you experience difficulties when speaking with one other person? (1) almost never or never- (5) practically always or always |
|  | Speech, Spatial, and Qualities of Hearing Scale (SSQ) | Gatehouse, S., & Noble, W. (2004). The speech, spatial and qualities of hearing scale (SSQ). *International journal of audiology*, *43*(2), 85-99. | 50 | 10 point Likert scales. Additional varied rating scales | 3 domains: speech, spatial, qualities | Can you easily have a conversation on the telephone? (1) not at all- (10) perfectly. |
|  | Subjective hearing capacity | Wahl, H. W., Heyl, V., & Schilling, O. (2012). Robustness of personality and affect relations under chronic conditions: The case of age-related vision and hearing impairment. *The Journals of Gerontology. Series B, Psychological Sciences and Social Sciences*, *67*(6), 687–696. https://doi.org/10.1093/geronb/gbs002 | 1 | 5 point likert scale. | 1 domain: Hearing capacity | How would you rate your hearing capacity? (1) very good- (5) very bad |
|  | Subjective vision capacity | Wahl, H. W., Heyl, V., & Schilling, O. (2012). Robustness of personality and affect relations under chronic conditions: The case of age-related vision and hearing impairment. *The Journals of Gerontology. Series B, Psychological Sciences and Social Sciences*, *67*(6), 687–696. https://doi.org/10.1093/geronb/gbs002 | 1 | 5 point likert scale. | 1 domain: vision capacity | How would you rate your vision capacity? (1) very good- (5) very bad |

**Supplementary S4:** Administration of included tools

^#^Information about this tool is derived from a secondary source, as the original source was unavailable/did not contain a study addressing the communication support needs and preferences of older adults.

|  | **Tool** | **Citation of tool** | **Self/**  **observer reported** | **Interview/**  **questionnaire/**  **Observations** | **In person/**  **online** | **Electronic/pencil paper** | **Administrator** | **Usability** | **Duration** |
| --- | --- | --- | --- | --- | --- | --- | --- | --- | --- |
|  | ASHA Functional Assessment of Communication Skills for Adults | Frattali, C.M., Thompson, C.K., Holland, A.L., Wohl, C.B., Wenck, C.J., Slater, S.C. & Paul, D. (2017) *American Speech-Language-Hearing Association Functional Assessment of Communication Skills for Adults (ASHA FACS).* https://apps.asha.org/eweb/OLSDynamicPage.aspx?Webcode=olsdetails&title=American+Speech-Language-Hearing+Association+Functional+Assessment+of+Communication+Skills+for+Adults+(ASHA+FACS) | Observer-reported^#^ | Observations^#^ | In person^#^ | Not reported^#^ | Researchers/  clinicians | Specialist | 20 minutes |
|  | ASHA Quality of Communication Life Scale | Paul, D.R, Frattali, C.M., Holland, A.L., Thompson, C.K., Caperton, C.J., Slater, S.C. (2004) *Quality of Communication Life Scale (ASHA QCL)* https://apps.asha.org/eweb/OLSDynamicPage.aspx?Webcode=olsdetails&title=Quality+of+Communication+Life+Scale+(ASHA+QCL) | Self-reported^#^ | Questionnaire^#^ | Not reported^#^ | Not reported^#^ | Researchers/  clinicians | Non-specialist | 15 minutes |
|  | Behavioural Assessment Scale (BAS)- communication subscale | Ritchie, K., & Ledesert, B. (1991). The measurement of incapacity in the severely demented elderly: The validation of a behavioural assessment scale. *International Journal of Geriatric Psychiatry*, *6*(4), 217–226. https://doi.org/10.1002/gps.930060406 | Observer-reported | Observations | In person | Not reported | Nursing staff or caretakers | Non-specialist | Not reported |
|  | Camberwell Assessment of Need for the Elderly (CANE) questionnaire | Reynolds, T., Thornicroft, G., Abas, M., Woods, B., Hoe, J., Leese, M., & Orrell, M. (2000). Camberwell Assessment of Need for the Elderly (CANE): Development, validity and reliability. British Journal of Psychiatry, 176(5), 444–452. https://doi.org/10.1192/bjp.176.5.444 | Self- and observer-report. | Questionnaire rated by patient, carer, and staff (non-specialist) | Not reported | Not reported | Researchers/  clinicians | Non-specialist | Within 30 minutes |
|  | Communication Behavior in People with Dementia in Ambulant Settings (CODEMamb) | Knebel, M., Haberstroh, J., Kümmel, A., Pantel, J., & Schröder, J. (2016). CODEM amb - an observational communication behavior assessment tool for use in ambulatory dementia care. *Aging & Mental Health*, *20*(12), 1286–1296. https://doi.org/10.1080/13607863.2015.1075959 | Observer-reported | Observations | Not reported | Not reported | Researchers/  clinicians | Non-specialist | 3 minutes |
|  | Montreal Evaluation of Communication Questionnaire for Use in Long Term Care (MECQ-LTC) | Le Dorze, G., Julien, M., Genereux, S., Larfeuil, C., Navennec, C., Laporte, D., & Champagne, C. (2000). The development of a procedure for the evaluation of communication occurring between residents in long-term care and their caregivers. *Aphasiology*, *14*(1), 17-51. https://doi.org/10.1080/026870300401586 | Observer-reported | Interview, observations | In person | Not reported | Caregivers: nurses, orderlies, professionals, students, volunteers | Specialist | 30 minutes |
|  | Communication Behavior in Dementia (CODEM) | Kuemmel, A., Haberstroh, J., & Pantel, J. (2014). CODEM Instrument: Developing a Tool to Assess Communication Behaviour in Dementia. GeroPsych, 27(1), 23–31. https://doi.org/10.1024/1662-9647/a000100 | Observer-reported | Observations | In person | Not reported | Researchers/  clinicians | Non-specialist | 3 minutes |
|  | Communication checklist | Dodd, B., Worrall, L., & Hickson, L. (1990). *Communication: a guide for residential care staff*. A.G.P.S. | Observer-reported | Observations | N/A | Not reported | Aged care staff | Non-specialist | Not reported |
|  | Communication difficulty of home-bound older adult | Rippy, J., Dancer, J., Pryor, R., & Stamper, J. (1986). A Field Experience: Rating the Communication Difficulty of Home-Bound Older Persons. *Home Health Care Services Quarterly*, *6*(3), 33–47. https://doi.org/10.1300/J027v06n03_04 | Observer-reported | Observations | In person | Not reported | Researchers/  clinicians | Non-specialist | Not reported |
|  | Communication plans | Généreux, S., Julien, M., Larfeuil, C., Lavoie, V., Soucy, O., & Le Dorze, G. (2004). Using communication plans to facilitate interactions with communication-impaired persons residing in long-term care institutions. Aphasiology, 18(12), 1161–1175. https://doi.org/10.1080/02687030444000507 | Self & observer-reported | Interview | Not reported | Not reported | Researchers/  clinicians | Specialist | Not reported |
|  | Communication Profile for the Hearing Impaired (CPHI)^#^ | Demorest, M. E., & Erdman, S. A. (1987). Development of the Communication Profile for the Hearing Impaired. The Journal of Speech and Hearing Disorders, 52(2), 129–143. https://doi.org/10.1044/jshd.5202.129 | Self-reported^#^ | Questionnaire completed by older adult^#^ | In person^#^ | Paper and pencil^#^ | Researchers/  clinicians | Non-specialist | 30-45 minutes |
|  | Communication Self-Assessment Scales for Older Adults (CSOA) | Kaplan, H., Bally, S., Brandt, F., Busacco, D., & Pray, J. (1997). Communication Scale for Older Adults (CSOA). *Journal of the American Academy of Audiology*, *8*(3), 203–217. | Self-reported | Questionnaire completed by older adult | In person | Pencil/paper based | Researchers/  clinicians | Non-specialist | Not reported |
|  | Communication‐support needs assessment tool for dementia (CoSNAT‐D) | Krein, L., Jeon, Y., Miller Amberber, A., & Fethney, J. (2022). Communication support needs assessment in dementia (CoSNAT‐D): An international content validation study. Health & Social Care in the Community, 30(6), e4745–e4757. https://doi.org/10.1111/hsc.13881 | Self-reported | Questionnaire completed by older adult/support person | N/A | N/A | Self-administered/  Support persons of people with dementia | Non-specialist | Not reported |
|  | Everyday Digital Literacy Questionnaire | Choi, J. Y., Choi, S., Song, K., Baek, J., Kim, H., Choi, M., Kim, Y., Chu, S. H., & Shin, J. (2023). Everyday Digital Literacy Questionnaire for Older Adults: Instrument Development and Validation Study. *JMIR. Journal of Medical Internet Research/Journal of Medical Internet Research*, *25*(5), e51616–e51616. https://doi.org/10.2196/51616 | Self-reported | Questionnaire completed by older adult | In person | Electronic using tablet | Researchers/  clinicians | Non-specialist | Not reported |
|  | Experienced Communication in Dementia (ECD-C & ECD-P) | Olthof-Nefkens, M. W. L. J., Derksen, E. W. C., Lambregts, B., de Swart, B. J. M., Nijhuis-Van der Sanden, M. W. G., & Kalf, J. G. (2023). Clinimetric Evaluation of the Experienced Communication in Dementia Questionnaire. The Gerontologist, 63(1), 40–51. https://doi.org/10.1093/geront/gnab187 | Self-reported | Questionnaire completed by older adult/support person | In person | Not reported | Researchers/  clinicians | Non-specialist | 10 minutes |
|  | Hearing Attitudes to Rehabilitation Questionnaire (HARQ) | Hallam, R. S., & Brooks, D. N. (1996). Development of the Hearing Attitudes in Rehabilitation Questionnaire (HARQ). British Journal of Audiology, 30(3), 199–213. https://doi.org/10.3109/03005369609079040 | Self-reported^#^ | Interview with older adult^#^ | In person^#^ | Not reported^#^ | Researchers/  clinicians | Non-specialist | Not reported |
|  | Hearing Handicap Inventory for the Elderly (HHIE)-short | Ventry, I. M., & Weinstein, B. E. (1982). The Hearing Handicap Inventory for the Elderly: a New Tool. *Ear and Hearing*, *3*(3), 128–134. https://doi.org/10.1097/00003446-198205000-00006 | Self-reported | Interview with older adult | In person | Not reported | Researchers/  clinicians | Non-specialist | 10 Minutes |
|  | Hearing Handicap Questionnaire (HHQ) | Gatehouse, S., & Noble, W. (2004). The Speech, Spatial and Qualities of Hearing Scale (SSQ). International Journal of Audiology, 43(2), 85–99. https://doi.org/10.1080/14992020400050014 | Self-reported^#^ | Interview with older adult^#^ | In person^#^ | Not reported^#^ | Researchers/  clinicians | Non-specialist | Not reported |
|  | InterRAI Community Health Assessment (CHA) | Morris, J.N, Berg, K., Bjorkgren, M, Dedercq, A., Finne-Soveri, H., Fries, B.E., Frijters, D., Gilgen, R., Gray, L., Henrard, J., Hirdes, J.P., Ljunggren, G., Nonemaker, S., Steel, K., Szczerbinska. K. (2010). InterRAI Community Health (CHA) Assessment Form and User’s Manual Version 9.1 https://catalog.interrai.org/products/978-1-936065-17-2 | Self-reported^#^ | Interview with older adult, observations^#^ | In person^#^ | Not reported^#^ | Researchers/  clinicians | Non-specialist | 90 minutes |
|  | Life-Worldly Communication Scale | Fukaya, Y., Kawaguchi, M., Okabe, M., Koyama, S., & Kitamura, T. (2024). Development of the Life-Worldly Communication Scale to Improve Quality of Life: Daily Conversation as Care for Older Adults. *Health Communication*, *39*(9), 1727–1737. https://doi.org/10.1080/10410236.2023.2233180 | Self-reported | Questionnaire completed by older adult | In person | Not reported | Researchers/  clinicians | Non-specialist | Not reported |
|  | Low Vision Visual Functioning Questionnaire (LV-VFQ)-short | The National Eye Institute 25-Item Visual Function Questionnaire (VFQ-25) Version 2000 https://www.rand.org/content/dam/rand/www/external/health/surveys_tools/vfq/vfq25_manual.pdf | Self-reported^#^ | Interview with older adult^#^ | Not reported^#^ | Not reported^#^ | Researchers/  clinicians | Non-specialist | Not reported |
|  | Modified communication environment assessment and planning guide | Hickson, L., Worrall, L., Wilson, J., Tilse, C., & Setterlund, D. (2005). Evaluating communication for resident participation in an aged care facility. *Advances in Speech-Language Pathology*, *7*(4), 245–257. https://doi.org/10.1080/14417040500337047 | Observer-reported | Observations | In person | Not reported | Researchers/  clinicians | Non-specialist | Not reported |
|  | Quantified Denver Scale of Communicative Function (QDS)- short | Tuley, M. R., Mulrow, C. D., Aguilar, C., & Velez, R. (1990). A Critical Reevaluation of the Quantified Denver Scale of Communication Function. *Ear and Hearing*, *11*(1), 56–61. https://doi.org/10.1097/00003446-199002000-00011 | Self-reported | Questionnaire completed by older adult | Not reported | Not reported | Researchers/  clinicians | Non-specialist | Not reported |
|  | Questionnaire of communication | Hickson, L., Worrall, L., Wilson, J., Tilse, C., & Setterlund, D. (2005). Evaluating communication for resident participation in an aged care facility. *Advances in Speech-Language Pathology*, *7*(4), 245–257. https://doi.org/10.1080/14417040500337047 | Self-reported | Interview with older adult | In person | Not reported | Researchers/  clinicians | Non-specialist | Not reported |
|  | Quick reference communication guide | Vermilya, S., & Stevens, L. C. (2012). A Guide for Enhancing Patient and Caregiver Communication. *Perspectives on Augmentative and Alternative Communication*, *21*(2), 32–36. https://doi.org/10.1044/aac21.2.32 | Observer-reported | Observations | In person | Not reported | Researchers/  clinicians | Non-specialist | Not reported |
|  | Self-Assessment/  significant other of Communication (SAC & SOAC) | Schow, R. L., & Nerbonne, M. A. (1982). Communication Screening Profile: Use with Elderly Clients. *Ear and Hearing*, *3*(3), 135–147. https://doi.org/10.1097/00003446-198205000-00007 | Self-reported | Questionnaire completed by older adult/support person | In person | Not reported | Self-administered/  Significant others | Non-specialist | Not reported |
|  | Speech, Spatial, and Qualities of Hearing Scale (SSQ) | Gatehouse, S., & Noble, W. (2004). The speech, spatial and qualities of hearing scale (SSQ). *International journal of audiology*, *43*(2), 85-99. | Self-reported | Interview with older adult | Not reported | Not reported | Researchers/  clinicians | Non-specialist | Not reported |
|  | Subjective hearing capacity | Wahl, H. W., Heyl, V., & Schilling, O. (2012). Robustness of personality and affect relations under chronic conditions: The case of age-related vision and hearing impairment. *The Journals of Gerontology. Series B, Psychological Sciences and Social Sciences*, *67*(6), 687–696. https://doi.org/10.1093/geronb/gbs002 | Self-reported | Interview with older adult | In person | Not reported | Researchers/  clinicians | Non-specialist | Not reported |
|  | Subjective vision capacity | Wahl, H. W., Heyl, V., & Schilling, O. (2012). Robustness of personality and affect relations under chronic conditions: The case of age-related vision and hearing impairment. *The Journals of Gerontology. Series B, Psychological Sciences and Social Sciences*, *67*(6), 687–696. https://doi.org/10.1093/geronb/gbs002 | Self-reported | Interview with older adult | In person | Not reported | Researchers/  clinicians | Non-specialist | Not reported |

Supplementary S4: A list of studies identified in this review that describe profiling tools.

|  | Tool | Reference |
| --- | --- | --- |
|  | ASHA Functional Assessment of Communication Skills for Adults^#^ | Davidson, B., Worrall, L., & Hickson, L. (2003). Identifying the communication activities of older people with aphasia: Evidence from naturalistic observation. Aphasiology, 17(3), 243-264. |
|  | ASHA Quality of Communication Life Scale^#^ | Lee, H., & Ha, J. (2024). Social isolation and its influencing factors among age-related hearing loss patients A cross-sectional study. Medicine, 103(4), E36766-e36766. https://doi.org/10.1097/MD.0000000000036766 |
|  | Behavioural Assessment Scale (BAS)- communication subscale | Ritchie, K., & Ledesert, B. (1991). The measurement of incapacity in the severely demented elderly: The validation of a behavioural assessment scale. *International Journal of Geriatric Psychiatry*, *6*(4), 217–226. https://doi.org/10.1002/gps.930060406 |
|  | Camberwell Assessment of Need for the Elderly (CANE) questionnaire | Reynolds, T., Thornicroft, G., Abas, M., Woods, B., Hoe, J., Leese, M., & Orrell, M. (2000). Camberwell Assessment of Need for the Elderly (CANE): Development, validity and reliability. British Journal of Psychiatry, 176(5), 444–452. https://doi.org/10.1192/bjp.176.5.444 |
|  | Communication Behavior in People with Dementia in Ambulant Settings (CODEMamb) | Knebel, M., Haberstroh, J., Kümmel, A., Pantel, J., & Schröder, J. (2016). CODEM amb - an observational communication behavior assessment tool for use in ambulatory dementia care. *Aging & Mental Health*, *20*(12), 1286–1296. https://doi.org/10.1080/13607863.2015.1075959 |
|  | Montreal Evaluation of Communication Questionnaire for Use in Long Term Care (MECQ-LTC) | Le Dorze, G., Julien, M., Genereux, S., Larfeuil, C., Navennec, C., Laporte, D., & Champagne, C. (2000). The development of a procedure for the evaluation of communication occurring between residents in long-term care and their caregivers. *Aphasiology*, *14*(1), 17-51. https://doi.org/10.1080/026870300401586 |
|  | Communication Behaviour in Dementia (CODEM) | Kuemmel, A., Haberstroh, J., & Pantel, J. (2014). CODEM Instrument: Developing a Tool to Assess Communication Behaviour in Dementia. GeroPsych, 27(1), 23–31. https://doi.org/10.1024/1662-9647/a000100 |
|  | Communication checklist | Dodd, B., Worrall, L., & Hickson, L. (1990). *Communication: a guide for residential care staff*. A.G.P.S. |
|  | Communication difficulty of home-bound older adult | Rippy, J., Dancer, J., Pryor, R., & Stamper, J. (1986). A Field Experience: Rating the Communication Difficulty of Home-Bound Older Persons. *Home Health Care Services Quarterly*, *6*(3), 33–47. https://doi.org/10.1300/J027v06n03_04 |
|  | Communication plans | Généreux, S., Julien, M., Larfeuil, C., Lavoie, V., Soucy, O., & Le Dorze, G. (2004). Using communication plans to facilitate interactions with communication-impaired persons residing in long-term care institutions. Aphasiology, 18(12), 1161–1175. https://doi.org/10.1080/02687030444000507 |
|  | Communication Profile for the Hearing Impaired (CPHI) ^#^ | Garstecki, D. C., & Erler, S. F. (1999). Older Adult Performance on the Communication Profile for the Hearing Impaired: Gender Difference. Journal of Speech, Language, and Hearing Research, 42(4), 785–796. https://doi.org/10.1044/jslhr.4204.785 |
|  | Communication Self-Assessment Scales for Older Adults (CSOA) | Kaplan, H., Bally, S., Brandt, F., Busacco, D., & Pray, J. (1997). Communication Scale for Older Adults (CSOA). *Journal of the American Academy of Audiology*, *8*(3), 203–217. |
|  | Communication‐support needs assessment tool for dementia (CoSNAT‐D) | Krein, L., Jeon, Y., Miller Amberber, A., & Fethney, J. (2022). Communication support needs assessment in dementia (CoSNAT‐D): An international content validation study. Health & Social Care in the Community, 30(6), e4745–e4757. https://doi.org/10.1111/hsc.13881 |
|  | Everyday Digital Literacy Questionnaire | Choi, J. Y., Choi, S., Song, K., Baek, J., Kim, H., Choi, M., Kim, Y., Chu, S. H., & Shin, J. (2023). Everyday Digital Literacy Questionnaire for Older Adults: Instrument Development and Validation Study. *JMIR. Journal of Medical Internet Research/Journal of Medical Internet Research*, *25*(5), e51616–e51616. https://doi.org/10.2196/51616 |
|  | Experienced Communication in Dementia (ECD-C) caregiver version/Experienced Communication in Dementia (ECD-P) patient version | Olthof-Nefkens, M. W. L. J., Derksen, E. W. C., Lambregts, B., de Swart, B. J. M., Nijhuis-Van der Sanden, M. W. G., & Kalf, J. G. (2023). Clinimetric Evaluation of the Experienced Communication in Dementia Questionnaire. The Gerontologist, 63(1), 40–51. https://doi.org/10.1093/geront/gnab187 |
|  | Hearing Attitudes to Rehabilitation Questionnaire (HARQ) ^#^ | Hickson, L., Worrall, L., & Scarinci, N. (2007). A Randomized Controlled Trial Evaluating the Active Communication Education Program for Older People with Hearing Impairment. Ear and Hearing, 28(2), 212–230. https://doi.org/10.1097/AUD.0b013e31803126c8 |
|  | Hearing Handicap Inventory for the Elderly (HHIE)-short | Ventry, I. M., & Weinstein, B. E. (1982). The Hearing Handicap Inventory for the Elderly: a New Tool. *Ear and Hearing*, *3*(3), 128–134. https://doi.org/10.1097/00003446-198205000-00006 |
|  | Hearing Handicap Questionnaire (HHQ) ^#^ | Hickson, L., Worrall, L., & Scarinci, N. (2007). A Randomized Controlled Trial Evaluating the Active Communication Education Program for Older People with Hearing Impairment. Ear and Hearing, 28(2), 212–230. https://doi.org/10.1097/AUD.0b013e31803126c8 |
|  | InterRAI Community Health Assessment (CHA)^#^ | Urqueta Alfaro, A., Guthrie, D. M., Phillips, N. A., Pichora-Fuller, M. K., Mick, P., McGraw, C., & Wittich, W. (2019). Detection of vision and/or hearing loss using the interRAI Community Health Assessment aligns well with common behavioral vision/hearing measurements. *PLoS One*, *14*(10), e0223123. |
|  | Life-Worldly Communication Scale | Fukaya, Y., Kawaguchi, M., Okabe, M., Koyama, S., & Kitamura, T. (2024). Development of the Life-Worldly Communication Scale to Improve Quality of Life: Daily Conversation as Care for Older Adults. *Health Communication*, *39*(9), 1727–1737. https://doi.org/10.1080/10410236.2023.2233180 |
|  | Low Vision Visual Functioning Questionnaire (LV-VFQ)-short^#^ | Leroi, I., Simkin, Z., Hooper, E., Wolski, L., Abrams, H., Armitage, C. J., Camacho, E., Charalambous, A. P., Collin, F., Constantinidou, F., Dawes, P., Elliott, R., Falkingham, S., Frison, E., Hann, M., Helmer, C., Himmelsbach, I., Hussain, H., Marié, S., … Yeung, W. K. (2020). Impact of an intervention to support hearing and vision in dementia: The SENSE‐Cog Field Trial. International Journal of Geriatric Psychiatry, 35(4), 348–357. https://doi.org/10.1002/gps.5231 |
|  | Modified communication environment assessment and planning guide | Hickson, L., Worrall, L., Wilson, J., Tilse, C., & Setterlund, D. (2005). Evaluating communication for resident participation in an aged care facility. *Advances in Speech-Language Pathology*, *7*(4), 245–257. https://doi.org/10.1080/14417040500337047 |
|  | Quantified Denver Scale of Communicative Function (QDS)- short | Tuley, M. R., Mulrow, C. D., Aguilar, C., & Velez, R. (1990). A Critical Reevaluation of the Quantified Denver Scale of Communication Function. *Ear and Hearing*, *11*(1), 56–61. https://doi.org/10.1097/00003446-199002000-00011 |
|  | Questionnaire of communication | Hickson, L., Worrall, L., Wilson, J., Tilse, C., & Setterlund, D. (2005). Evaluating communication for resident participation in an aged care facility. *Advances in Speech-Language Pathology*, *7*(4), 245–257. https://doi.org/10.1080/14417040500337047 |
|  | Quick reference communication guide | Vermilya, S., & Stevens, L. C. (2012). A Guide for Enhancing Patient and Caregiver Communication. *Perspectives on Augmentative and Alternative Communication*, *21*(2), 32–36. https://doi.org/10.1044/aac21.2.32 |
|  | Self-Assessment of Communication (SAC)/ Significant other assessment of Communication (SOAC) | Schow, R. L., & Nerbonne, M. A. (1982). Communication Screening Profile: Use with Elderly Clients. *Ear and Hearing*, *3*(3), 135–147. https://doi.org/10.1097/00003446-198205000-00007 |
|  | Speech, Spatial, and Qualities of Hearing Scale (SSQ) | Gatehouse, S., & Noble, W. (2004). The speech, spatial and qualities of hearing scale (SSQ). *International journal of audiology*, *43*(2), 85-99. |
|  | Subjective hearing capacity | Wahl, H. W., Heyl, V., & Schilling, O. (2012). Robustness of personality and affect relations under chronic conditions: The case of age-related vision and hearing impairment. *The Journals of Gerontology. Series B, Psychological Sciences and Social Sciences*, *67*(6), 687–696. https://doi.org/10.1093/geronb/gbs002 |
|  | Subjective vision capacity | Wahl, H. W., Heyl, V., & Schilling, O. (2012). Robustness of personality and affect relations under chronic conditions: The case of age-related vision and hearing impairment. *The Journals of Gerontology. Series B, Psychological Sciences and Social Sciences*, *67*(6), 687–696. https://doi.org/10.1093/geronb/gbs002 |

^#^Information about this tool is derived from a secondary source, as the original source was unavailable/did not contain a study addressing the communication support needs and preferences of older adults.

Supplementary S5: PRISMA Sc-R Checklist

| **SECTION** | **ITEM** | **PRISMA-ScR CHECKLIST ITEM** | **REPORTED ON PAGE #** |
| --- | --- | --- | --- |
| **TITLE** | | | |
| Title | 1 | Identify the report as a scoping review. | p. 1 |
| **ABSTRACT** | | | |
| Structured summary | 2 | Provide a structured summary that includes (as applicable): background, objectives, eligibility criteria, sources of evidence, charting methods, results, and conclusions that relate to the review questions and objectives. | p.2 |
| **INTRODUCTION** | | | |
| Rationale | 3 | Describe the rationale for the review in the context of what is already known. Explain why the review questions/objectives lend themselves to a scoping review approach. | p.3-4 |
| Objectives | 4 | Provide an explicit statement of the questions and objectives being addressed with reference to their key elements (e.g., population or participants, concepts, and context) or other relevant key elements used to conceptualize the review questions and/or objectives. | p. 3-4 |
| **METHODS** | | | |
| Protocol and registration | 5 | Indicate whether a review protocol exists; state if and where it can be accessed (e.g., a Web address); and if available, provide registration information, including the registration number. | p.4 |
| Eligibility criteria | 6 | Specify characteristics of the sources of evidence used as eligibility criteria (e.g., years considered, language, and publication status), and provide a rationale. | p. 5, table 2 |
| Information sources* | 7 | Describe all information sources in the search (e.g., databases with dates of coverage and contact with authors to identify additional sources), as well as the date the most recent search was executed. | p. 5 |
| Search | 8 | Present the full electronic search strategy for at least 1 database, including any limits used, such that it could be repeated. | p. table 1, S1 |
| Selection of sources of evidence† | 9 | State the process for selecting sources of evidence (i.e., screening and eligibility) included in the scoping review. | p. 5-7 |
| Data charting process‡ | 10 | Describe the methods of charting data from the included sources of evidence (e.g., calibrated forms or forms that have been tested by the team before their use, and whether data charting was done independently or in duplicate) and any processes for obtaining and confirming data from investigators. | p. 6-7 |
| Data items | 11 | List and define all variables for which data were sought and any assumptions and simplifications made. | p. 6-7 |
| Critical appraisal of individual sources of evidence§ | 12 | If done, provide a rationale for conducting a critical appraisal of included sources of evidence; describe the methods used and how this information was used in any data synthesis (if appropriate). | N/A |
| Synthesis of results | 13 | Describe the methods of handling and summarizing the data that were charted. | p. 7 |
| **RESULTS** | | | |
| Selection of sources of evidence | 14 | Give numbers of sources of evidence screened, assessed for eligibility, and included in the review, with reasons for exclusions at each stage, ideally using a flow diagram. | p.7, figure 1 |
| Characteristics of sources of evidence | 15 | For each source of evidence, present characteristics for which data were charted and provide the citations. | Table 3, S2-3 |
| Critical appraisal within sources of evidence | 16 | If done, present data on critical appraisal of included sources of evidence (see item 12). | N/A |
| Results of individual sources of evidence | 17 | For each included source of evidence, present the relevant data that were charted that relate to the review questions and objectives. | Table 3, S2-5 |
| Synthesis of results | 18 | Summarize and/or present the charting results as they relate to the review questions and objectives. | p.7-11 |
| **DISCUSSION** | | | |
| Summary of evidence | 19 | Summarize the main results (including an overview of concepts, themes, and types of evidence available), link to the review questions and objectives, and consider the relevance to key groups. | p.11-13 |
| Limitations | 20 | Discuss the limitations of the scoping review process. | p.14 |
| Conclusions | 21 | Provide a general interpretation of the results with respect to the review questions and objectives, as well as potential implications and/or next steps. | p. 14 |
| **FUNDING** | | | |
| Funding | 22 | Describe sources of funding for the included sources of evidence, as well as sources of funding for the scoping review. Describe the role of the funders of the scoping review. | p. 15 |

JBI = Joanna Briggs Institute; PRISMA-ScR = Preferred Reporting Items for Systematic reviews and Meta-Analyses extension for Scoping Reviews.

* Where *sources of evidence* (see second footnote) are compiled from, such as bibliographic databases, social media platforms, and Web sites.

† A more inclusive/heterogeneous term used to account for the different types of evidence or data sources (e.g., quantitative and/or qualitative research, expert opinion, and policy documents) that may be eligible in a scoping review as opposed to only studies. This is not to be confused with *information sources* (see first footnote).

‡ The frameworks by Arksey and O’Malley (6) and Levac and colleagues (7) and the JBI guidance (4, 5) refer to the process of data extraction in a scoping review as data charting*.*

§ The process of systematically examining research evidence to assess its validity, results, and relevance before using it to inform a decision. This term is used for items 12 and 19 instead of "risk of bias" (which is more applicable to systematic reviews of interventions) to include and acknowledge the various sources of evidence that may be used in a scoping review (e.g., quantitative and/or qualitative research, expert opinion, and policy document).

*From:* Tricco AC, Lillie E, Zarin W, O'Brien KK, Colquhoun H, Levac D, et al. PRISMA Extension for Scoping Reviews (PRISMAScR): Checklist and Explanation. Ann Intern Med. 2018;169:467–473. [doi: 10.7326/M18-0850](http://annals.org/aim/fullarticle/2700389/prisma-extension-scoping-reviews-prisma-scr-checklist-explanation).
